# Impact of Pollution on Cancer: A Systematic Review and Meta-Analysis with Focus on Air Pollution

**DOI:** 10.3390/ijerph23040429

**Published:** 2026-03-30

**Authors:** Sagar Sharma Timilsina, Tilak Bhusal, Avishek Choudhury

**Affiliations:** Department of Industrial and Management Systems Engineering, West Virginia University, Morgantown, WV 26506, USA; ss00277@mix.wvu.edu (S.S.T.); tb00089@mix.wvu.edu (T.B.)

**Keywords:** air pollution, particulate matter, water pollution, land pollution, cancer, heavy metals

## Abstract

**Highlights:**

**Public health relevance—How does this work relate to a public health issue?**

- Environmental pollution is a major global public health problem and is increasingly associated with cancer incidence, morbidity, and premature mortality.
- This study synthesizes existing evidence on the associations between air, water, and land pollution with cancer risk across populations.

**Public health significance—Why is this work of significance to public health?**

- This work provides consolidated evidence that multiple pollutants, particularly particulate matter, are associated with increased risk of cancer.
- The findings present substantial disparities in pollution-related cancer research and burden across pollution types, socioeconomic groups, and regions.

**Public health implications—What are the key implications or messages for practitioners, policy makers and/or researchers in public health?**

- Strengthened pollution control policies, stricter implementation of existing regulations, and targeted interventions in high-risk and vulnerable populations are needed to reduce cancer burden.
- Future research should focus on underrepresented regions and non-air pollution pathways, while incorporating socioeconomic and demographic disparities in cancer risk assessment.

**Abstract:**

Pollution remains a major global public health concern increasingly associated with cancer incidence. This systematic review and meta-analyses examined the association between cancer risk and pollution across air, water, and land following the PRISMA guidelines. From 26,367 records initially identified in PubMed, Web of Science, and Scopus (January 2014–June 2025), 168 studies met the eligibility criteria. Meta-analyses conducted on 11 groups of studies revealed significant associations of lung cancer with fine particulate matter (HR_pooled_ = 1.347; 95% CI: 1.158–1.536), black carbon (HR_pooled_ = 1.096; 95% CI: 1.014–1.179) and ozone (HR_pooled_ = 0.941; 95% CI: 0.908–0.975), and breast cancer with nitrogen dioxide (HR_pooled_ = 1.064; 95% CI: 1.011–1.117). The association of ozone with cancer risks was inconsistent. While 155 studies reported on cancer risks from air pollution, only 10 studies focused on water pollutants and two on land pollutants, primarily heavy metals. Also, 79% of reviewed studies originated from only six high-income countries. The findings suggest that while particulate matter is a consistent risk factor, the global evidence base remains imbalanced based on pollution type and economic status of countries. Addressing these data gaps through targeted research in underrepresented regions and prioritizing the reduction of exposure to identified carcinogenic pollutants could reduce the global cancer burden.

## 1. Introduction

In 2019, pollution was found to be responsible for 9 million premature deaths [1], and it remains a global public health hazard. According to the United Nations Statistics Division, “pollution is defined as the presence of substances and/or heat in environmental media (air, water, land) whose nature, location, or quantity produces undesirable environmental effects” [2]. In simpler terms, anything unwanted added to the environment is pollution. Among the multiple types of pollution, air, water, and land pollution are considered the major types.

According to the World Health Organization (WHO), about 99% of the global population is exposed to air pollutants exceeding their safety guidelines [3], with ambient air pollution being the deadliest form of pollution [4]. The associations between environmental pollutants with multiple public health issues are documented across all media [5,6,7,8,9,10,11,12]. Among them, outdoor air pollution and particulate matter have the most robust evidence supporting their role in cancer development, leading to their classification as known (Group 1) human carcinogens [13]. Lung cancer remains most closely and consistently related to these exposures [1]. The biological mechanisms primarily underlying this relationship include oxidative stress, chronic systemic inflammation, and DNA damage by reactive free radicals [14]. In contrast, the relationship of land and water contamination with cancer remains uncertain as exposure often depends on complex ingestion and dermal pathways that vary significantly by region [1,15].

Cancer is one of the most critical public health issues because of its life-threatening nature in most cases and substantial economic burden (if curable). It remains a major public health challenge worldwide, with incidence anticipated to rise greatly through 2050 [16]. This rise is expected mainly in lower-income countries. According to the International Agency for Research on Cancer (IARC), the global cancer incidence in 2022 was 19,764,999, and the global cancer mortality was 9,743,320 [17]. For the USA, the estimated new cancer cases in 2025 are 226,650, and the 5-year relative survival rate for cancer of any site is 69.9% for 2015 to 2021 [18] according to the National Cancer Institute (NCI). Furthermore, the risk of dying from cancer before the age of 75 years was 9.6% in a global perspective [17]. Thus, efforts to reduce cancer incidence and its global burden are necessary. As pollution exposure is one of the major factors found to be associated with cancer risks, researching the topic of pollution-related cancer is essential.

Furthermore, factors such as lower socioeconomic status, race, gender, etc., can play a significant role in dictating exposure to carcinogens, health care access, awareness, and many more. Poverty has been found to have a direct impact on cancer-specific outcomes [19]. Similarly, increasing unemployment was found to be associated with statistically significant increases in cancer mortality [20]. In addition, according to the NCI, African-Americans have a greater mortality rate than others for many cancer types, though not all [21]. Social factors are responsible for creating disparities in cancer, which makes it essential to conduct studies to assess the effects of social factors on cancer. So, our review has included studies discussing the impacts of socio-demographic and socioeconomic factors on cancer, too.

Existing reviews have either focused on a single pollution (or a single pollutant) and its association with cancers or on a pollution with a specific cancer. A comprehensive study investigating the association of all, air, water, and land pollution with cancer, along with the social factors, seems to be lacking. This review aims to fill this gap by systematically reviewing the existing literature that has studied the association of pollution and social factors with cancer and conducting meta-analyses using the available data in them wherever feasible (i.e., if the set criteria for the data are met). Specifically, this study seeks to assess how different pollutants across the air, water and land media along with different social factors are influencing human cancer risks. This systematic review, along with meta-analyses of the existing literature, will help to present the existing evidence of the association of different pollution and social factors with cancer across the human body effectively, clearly, and concisely.

## 2. Methods

### 2.1. Protocol Registration

This systematic review and meta-analysis follow the Preferred Reporting Items for Systematic Reviews and Meta-Analysis (PRISMA) guidelines [22]. The PRISMA checklist (Table S1) and flowchart have been used to facilitate the review process. Protocol registration was completed with the Open Science Framework (OSF) on 16 January 2025 [23].

### 2.2. Search Strategy

The peer-reviewed articles and conference proceedings were searched in the PubMed, Scopus, and Web of Science databases from January 2014 to June 2025 to identify the ones that met the eligibility criteria of this systematic literature review as mentioned in Section 2.3.1. The initial search was carried out on 7 December 2024, and the last update was done on 10 July 2025. The search terms were selected using a modified PECO (Population–Exposure–Control–Outcome) framework. The population was defined as the general human population without any restriction. Moreover, the exposure terms were selected to match the scope of this review, which was to cover the literature that studies the effect of different pollution, social, demographic, and economic factors on cancer. A specific control group was not pre-defined in the search query to ensure a comprehensive and inclusive retrieval of all relevant empirical studies, while the outcome was defined as any reported cancer. Based on the terminologies listed in Table 1, search queries were developed specifically for all three databases (PubMed, Scopus, and Web of Science). Queries were developed by combining the keywords with Boolean operators (AND/OR) to identify all the relevant studies matching the scope and inclusion/exclusion criteria of this review.

The search query used for the Web of Science (WoS) database is: “TS = (Cancer) AND TS = (Socio-economic OR economic OR economics OR demographic OR socio-demographic OR Hazard Risk) AND TS = (Air Pollution OR Water pollution OR Land Pollution OR Pollution OR hazardous waste OR Pollutants OR PM OR Particulate Matter OR Ozone OR O_3_ OR Diesel PM OR Lead OR Toxic waste)”.

For the other two databases, the same search query but formatted accordingly to the respective databases was used.

### 2.3. Study Selection and Quality Assurance

The search of the publication was followed by multiple layers of screening. Inclusion/exclusion criteria were set beforehand to facilitate the screening process.

#### 2.3.1. Inclusion Criteria

- Peer-reviewed articles and conference proceedings;
- Published from 2014 to June 2025;
- Published in English language;
- Empirical studies reporting determinants of cancer outcomes.

#### 2.3.2. Exclusion Criteria

- No cancer outcomes reported;
- Review articles;
- Theoretical framework;
- Dissertations and theses;
- Books and book chapters;
- Reports;
- Preprints;
- Editorials.

Before screening, the literature information was extracted in csv file format from each database using the available literature data download options. The extracted literature information from multiple databases was merged, and then duplicates were removed both manually and with the help of the duplicate-removing feature of Microsoft Excel [24]. Screening was manual and multiphase: title screening was completed first (studies related to cancer were separated from this screening). It was a manual process completed by going through the titles of each extracted publication and removing the ones that did not meet the inclusion/exclusion criteria. It was followed by abstract screening, where the abstracts were reviewed for each of the publications that passed the title screening. Then, full paper screening was performed to reach the final volume of the literature for reviewing and data extraction. Each of the screening phases (title screening, abstract screening and full paper screening) was done by two reviewers simultaneously and independently. At the end of each phase, the selections were tallied and all discrepancies were resolved by discussion, requiring consensus from two senior researchers to ensure the control of selection bias before moving on to the next phase of screening. The selection process is documented in Table S2.

### 2.4. Quality Appraisal

For the quality appraisal purpose, the Mixed Methods Appraisal Tool (MMAT), version 2018 was used to assess the methodological quality of studies included in this systematic review as this tool was useful for assess both qualitative and quantitative evidence [25].

### 2.5. Data Extraction

Data extraction was completed on 10 July 2025. The country of origin, study design, study objectives, health outcomes, concerned contributing factors, and key findings were extracted from each of the reviewed papers.

Furthermore, to facilitate meta-analysis, sample sizes (if available), cancer types reported, exposure variables, effect sizes (Odds ratio, Hazard Ratio, and Risk Ratio) types along with their numerical values with upper control limit (UCL) and lower control limit (LCL), and *p*-value were also extracted. The data were compiled in Microsoft Excel [24].

### 2.6. Analysis

To visualize the various studies better, a Sankey diagram was plotted that linked the countries where studies originated, with the factors’ groups (air pollution, water pollution, and land pollution), followed by cancer groups. Different types of cancers are grouped based on the grouping used in the site recode ICD-0-3 2023 revision definition by the National Cancer Institute in Surveillance, Epidemiology, and End Results Program [26]. First, the qualitative review was conducted for all the selected studies, which was followed by a meta-analysis. Meta-analyses were conducted for each unique combination of cancer type, effect types, i.e., Odds ratio (OR)/Hazard Ratio (HR)/Risk Ratio (RR), and exposure variable, if at least 4 studies were reporting such and if those studies had met at least 3 of the 5 MMAT criteria. For example, a meta-analysis was done if at least 4 studies reported the HR of PM_2.5_ (fine particulate matter with a diameter less than 2.5 µm) exposure for brain cancer. The classical meta-analysis option in the JASP tool, version 0.19.3 [27] was used to conduct the meta-analysis. A common random effect model of meta-analysis, the DerSimonian and Laird method [28], was used due to the wide variation in sample sizes. The random-effects model was chosen because it explains the study variations better than any fixed effects method [29]. Combined effect sizes were determined with a 5% confidence interval. For doing the meta-analysis, standard errors (SE) were required, which were calculated from the control limits [28]: upper control limits (UCL) and lower control limits (LCL) using Equation (1).

SE = (ln(UCL) − ln(LCL))/3.92,(1)

For assessing the heterogeneity, I^2^, τ^2^, and τ was used. I^2^ describes the proportion of total variation attributable to heterogeneity, or the variance between studies in the meta-analysis. [30]. Furthermore, τ^2^ is the between-study variance and τ is the standard deviation. These values help to describe the dispersion in the true effect sizes of the studies. Statistical significance was interpreted based on the 95% confidence intervals, with estimates considered statistically significant only when the confidence interval did not include the null value and *p*-value less than 0.05. Finally, the forest plots of the studies were plotted.

## 3. Results

### 3.1. Search and Screening Results

**Figure 1:**
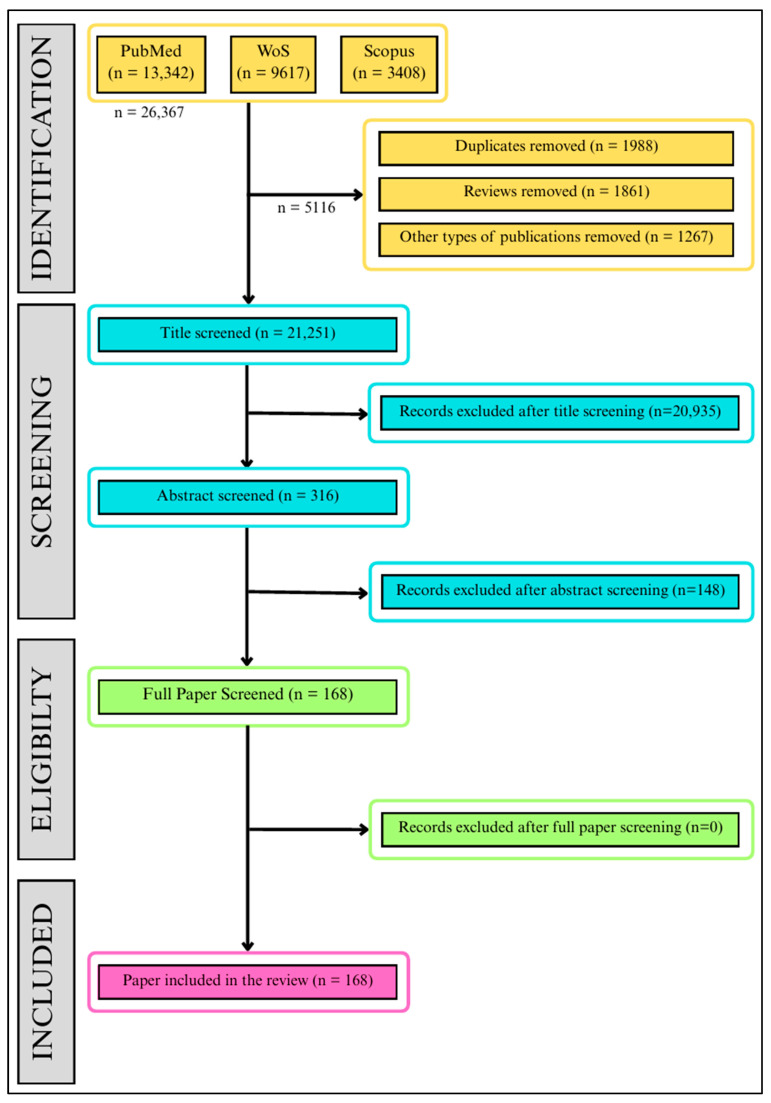
Preferred Reporting Items for Systematic Reviews and Meta-Analysis (PRISMA) flow chart presenting the selection process for the eligible publications to include in the systematic review.

The initial search had found 26,367 articles from across the three considered databases. The screening led to a reduction in this large volume to 168 papers for the final review. The final list of papers to review included only one [31] conference paper and all other peer-reviewed papers. The PRISMA flow diagram is shown in Figure 1.

### 3.2. Qualitative Review Results

**Figure 2:**
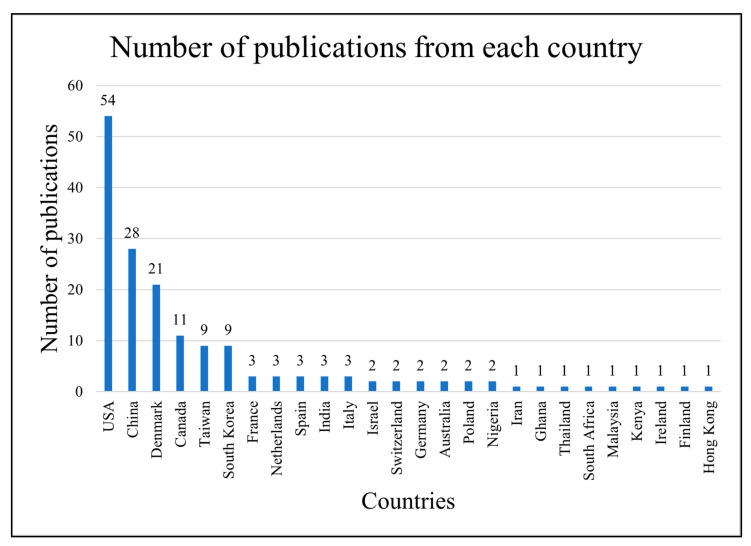
Distribution of publications from each country.

Among the reviewed papers, 54 studies (about one-third) were from the United States, 28 were from China, 21 were from Denmark, 11 were from Canada, 9 were from Taiwan, 9 were from South Korea, and so on. The distribution is shown in Figure 2 below. This wide variation in the geographic location of the studies shows that the review covered a diverse geography; however, with a great focus on high-income and developed countries.

**Figure 3:**
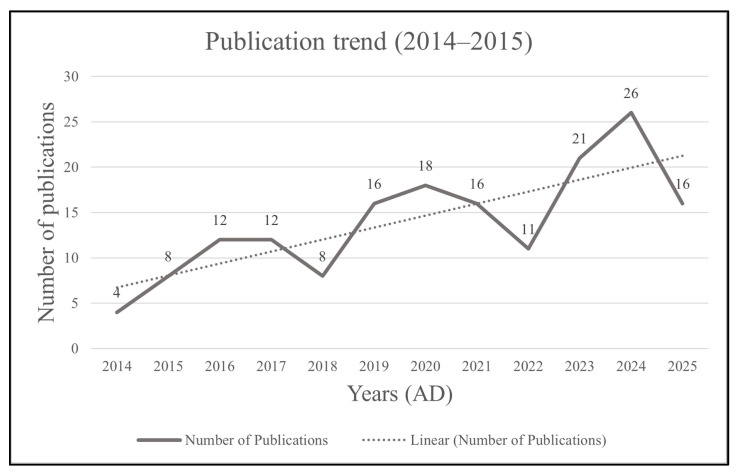
Publication trend from 2014 AD to 2025 AD.

In general, the trendline in Figure 3 shows that the number of publications each year seems to be rising, with some falls in 2018, 2021, and 2022. The highest number of publications was in the year 2024. Up to June 2025, there are already 16 studies. Thus, a spike in the number of yearly publications can be expected by the end of the year.

**Figure 4:**
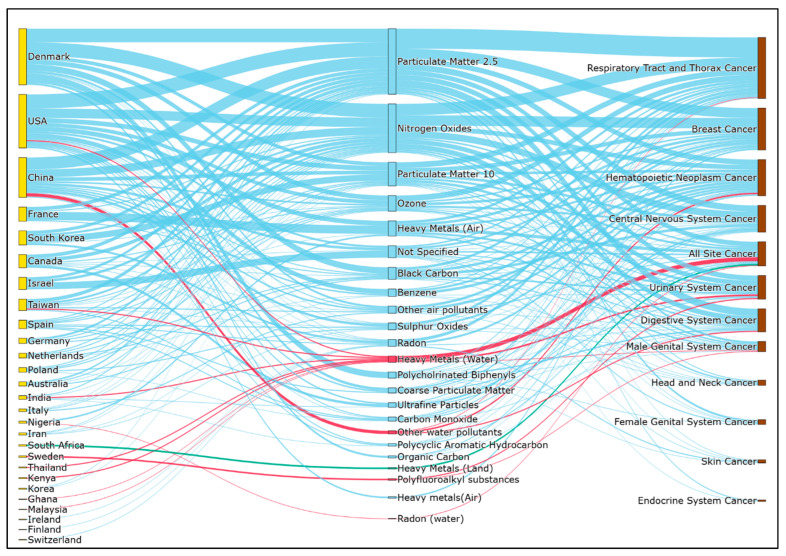
Sankey diagram showing the connection of pollutants, cancer groups and the countries where research is done. Yellow and brown nodes represent countries and cancer groups, respectively. Also, blue, red, and green flow lines and nodes show air, water, and land pollution, respectively.

In Figure 4, blue colored flow lines represent air pollution, red colored ones represent water pollution, and green colored flow lines represent land pollution. It can be seen that the greatest volume of research was focused on air pollution-related cancer research, and the USA, Denmark, and China are the major contributors. Within the air pollutants, fine particulate matter with a diameter less than 2.5 μm (PM_2.5_), nitrogen oxides (NO_x_), PM_10_ (particulate matter with a diameter less than 10 μm), and ozone (O_3_) are the most studied ones. These pollutants were related to a large variety of cancers, but mainly to respiratory tract and thorax cancer, breast cancer, hematopoietic neoplasm cancer, and central nervous system cancers. In addition, water pollution and land pollution also appear in the studies, but much less in comparison to air pollution. Among them, land pollution is the least frequently appearing category of pollution in the studies. In water pollution, heavy metals are the most considered pollutants.

#### 3.2.1. Air Pollution

Among the 168 studies reviewed, 155 reported on air pollution (Table S3). In these studies, the association of cancer risks with different air pollutants, including various types of particulate matter (ultra-fine particulate matter (UFP), PM_2.5_, PM_2.5-10_, and PM_10_), nitrogen oxides (NO_x_), heavy metals, and ozone (O_3_), has been studied. Increased exposure to these different pollutants has been found to increase the risk of different cancers, including breast cancer, lung cancer [32,33], and hematopoietic neoplasm cancers [34,35].

The association of PM_2.5_ with multiple cancers was found to be studied in 82 different studies; in 26 studies, its relation was studied with lung cancer. The effect sizes reported were found to be positively related to lung cancer in all of the studies and significantly in 16 of those studies [36,37,38,39,40,41,42,43,44,45,46,47,48,49,50,51]. These studies show that increased exposure to PM_2.5_ increases the lung cancer risks. In addition, six other studies also found increased lung cancer incidence linked to PM_2.5_ exposure [6,31,52,53,54,55]. Furthermore, a study done in China showed that the lung cancer mortality attributable to PM_2.5_ exposure increased by 4.11% annually [56]. However, a study from the USA discovered a non-significant association of PM_2.5_ with lung cancer in women who have never smoked [57]. Similarly, the association of fine particulate matter, i.e., PM_2.5_, was studied with breast cancer in 16 studies [32,33,36,58,59,60,61,62,63,64,65,66,67,68,69,70], and no negative association was observed with overall breast cancer. Four of the studies [33,36,69,70] reported statistically significant increased breast cancer risk with increased PM_2.5_ exposure. However, an inverse association, i.e., decreased risk of postmenopausal breast cancer, was observed with PM_2.5_ in a nurse cohort in the USA [65]. Furthermore, four studies [36,71,72,73] reported increased risk of “all site cancer” (specific cancer type not mentioned) with increased exposure to PM_2.5_. In addition, a study done in Lucknow, India, found that the reported excess lifetime cancer risk associated with indoor PM_2.5_ and UFP exposure exceeded permissible limits in young women [74].

The relation between PM_2.5_ and different types of central nervous system (CNS) cancer was examined in 10 of the studies [5,72,75,76,77,78,79,80,81,82]. However, only three of them found statistically significant increased cancer risks with increased PM_2.5_ exposure [76,78,81]. In addition, the association of fine particulate matter with liver cancer was studied in six studies [83,84,85,86,87,88], four [83,84,85,87] of which discovered significant positive associations, suggesting increased risk. Furthermore, increased PM_2.5_ exposure was found to have a significantly increase the risk of bladder cancer [36], prostate cancer [36], lymphoma [34], colorectal cancer [89,90], esophageal cancer [91], upper-aerodigestive tract cancer [92], laryngeal cancer [93], and oral cancer [94], leukemia [35], non-Hodgkin lymphoma [95], respiratory system cancer [96], and papillary thyroid cancer [93,97], pancreatic cancer [98,99] and ovarian cancer [100]. In addition, a Chinese study discovered increased mortality risk from ovarian cancer with increased PM_2.5_ exposure [101]. Furthermore, in a different study [102], the sulfur component of PM_2.5_ was found to increase the gastric cancer significantly. However, a few studies [103,104,105] did not find any significant association of cancer risks with fine particulate matter exposure.

It was discovered that the association of PM_10_ with lung cancer was studied in 11 different studies [37,47,51,61,106,107,108,109,110,111]. Among them, increased exposure to PM_10_ significantly increased the risk of lung cancer as a whole in two studies [37,107], and with adenocarcinoma [47] and squamous cell cancer [51] in one study each. Furthermore, a nationwide South Korean cohort study found that the increased exposure to PM_10_ increased the risk of urologic cancer, kidney cancer, and prostate cancer significantly [112].

Moreover, eight studies [58,61,62,63,64,65,70,113] were found to study the association of PM_10_ with breast cancer. However, a significant association was seen in only one study, where the risk of having breast cancer from PM_10_ exposure was found to be 1.99 times that of those without exposure in the UK [58]. Furthermore, a study done in Jharia coalfield in India, a coal mining area, discovered that the cancer risks from both PM_2.5_ and PM_10_ were found to exceed the United States Environmental Protection agency (USEPA) limit [114]. However, in a study done in Italy [115], a significant inverse association was discovered between PM_10_ and PM_2.5_ exposure with melanoma risk.

In the reviewed studies, nine studies [37,39,42,44,46,47,49,51,116] discovered a significantly increased risk of lung cancer with nitrogen dioxide (NO_2_). Among them, increased risk of adenocarcinoma was increased exposure to NO_2_ in three of the studies [39,42,47]. Also, studies done in the UK [37] and Israel [36] have found that the lung cancer risk increases with increased NO_x_ exposure. In addition, eleven studies [58,59,60,61,62,63,64,67,70,113,117] reported the hazard risk of NO_2_ with breast cancer, and among them, only two studies [59,64] found statistically significant increased risk with increased exposure. A nationwide Canadian study revealed that higher exposure to NO_2_ was associated with increased breast cancer risk in pre-menopausal women [116]. Furthermore, six studies studied the association of brain cancer with nitrogen oxides [5,76,77,79,80,81]. Among them, a study [81] from Denmark reported significant odds of getting meningioma with increased NO_2_ exposure. Also, increased exposure to NO_2_ increased the risk of leukemia [34], upper aero-digestive tract (UADT) cancer [92], prostate cancer [36,118], laryngeal cancer [119], Hodgkin lymphoma [120], and female uterine cancer [121]. Furthermore, the risk of bladder cancer and leukemia was discovered to significantly increase with the increased exposure to nitrogen oxides in Israel [36] and Tehran [122], respectively.

In this review, the association of ozone exposure and lung cancer was found to be studied in eight studies [39,40,43,44,46,67,111,123]. Among them, four of the studies [39,44,46,67] found that the overall lung cancer risk decreased with increased ozone exposure. Similar inverse association was discovered for squamous cell carcinoma [39], UADT cancer [92], liver cancer [83], and meningioma [81] with ozone exposure. However, a few studies discovered a significantly increased cancer risk with increased ozone exposure. In a UK study, prostate cancer risk was found to be increased with increased ozone exposure [118]. Similarly, in another study done in China, increased risk of lung cancer was found with ozone exposure [43].

Radon is another air pollutant whose association with cancer risks was studied in 12 studies. The relation of radon exposure with cancer risks was studied for different types of cancer, including lung cancer [44,124,125,126,127,128,129], leukemia [130,131], lymphoma [128], and skin cancers [132,133]. Among all these studies, increased radon exposure was discovered to significantly increase blood cancer risk [44] and lung cancer risk [124,125].

Exposure to airborne heavy metals was also found to increase various types of cancers. Cadmium exposure was found to have a significantly increased bladder cancer risk [134], lung cancer [135], and cancer as a whole [134]. Similarly, chromium was found to be increasing the risk of bladder cancer and cancer as a whole in a French cohort [134]. A US nationwide study discussed the association of chromium exposure with breast cancer; however, the association was not statistically significant [136]. Furthermore, lead exposure was found to significantly increase the risk of cancer in a French cohort [134] and the risk of meningioma [137] in Shanghai, China.

Furthermore, it has been found in studies that the increased exposure to other air pollutants, including black carbon (BC), carbon monoxide (CO), radon, sulfur dioxide (SO_2_), and UFPs, is also significantly increasing the risk of various cancers. Black carbon exposure was found to increase lung cancer risks [39,44,49], UADT cancer [92], breast cancer [33], malignant brain tumors [78], non-Hodgkin lymphoma [95], intracranial central nervous system (CNS) cancer [81], and cancer as a whole [71] in different studies. In addition, carbon monoxide (CO) exposure increased the risk of benign brain tumors [76], breast cancer [59], and adenocarcinoma (in the population below 75 years of age) [47]. However, the risk of adenocarcinoma was found to be reduced with CO exposure in the population aged greater than 75 years in a study conducted in a population from south-eastern Poland [47]. The same study discovered an increased risk of adenocarcinoma with sulphur dioxide (SO_2_) exposure. SO_2_ was significantly associated with lung cancer in another study done in the USA [138], too. It was also found to significantly increase the risk of esophageal cancer [139]. Moreover, six studies assessed the association of ultra-fine particle exposure with blood cancer [140], lung cancer [141,142], and brain cancer [75,143,144]. Among them, a study [143] discovered a statistically significant increased risk of brain cancer with increased UFP exposure in Toronto, Canada. Nearness to roads has also been linked with cancer risks. Air pollution from road proximity was found to be significantly associated with different cancers, such as blood cancer [145], CNS tumors [146], and breast cancer [147].

The air pollutant exposure score (APES) was found to be significantly associated with colorectal cancer [148] in the UK, and pesticide exposure was found to increase lymphoma risk in children from Georgia, USA [149]. One study found a significant positive correlation between phthalate esters and increased lifetime cancer risk exceeding the threshold of USEPA of 10^−6^ [150].

In addition, different studies have discovered increased risks of various cancers other various air pollutants. One Canadian study discovered such an association of bladder cancer risks with diesel exhaust [151]. Another study from Haifa Bay Area in Israel discovered an increased risk of breast cancer, leukemia, CNS cancer, melanoma, and thyroid cancer, significantly with industrial air pollution [152]. However, a Chinese study discovered an inverse relation between breast cancer and cervical cancer with soot exposure [153]. Whereas, another study done in Port Harcourt, Nigeria, found cancer risks from soot exposure within limits [154]. Other air pollutants that have been reported to increase cancer risks polycyclic aromatic hydrocarbons (PAHs) [155,156,157,158,159], industrial waste gas [160], volatile organic compounds (VOCs) [161,162], benzene [77,163,164,165], industry-related toxics [166], polychlorinated dibenzo-p-dioxins and dibenzofurans (PCDD/F) [167] household air pollutants [168,169], asbestos [170], polychlorinated biphenyls (PCBs) [171], methylene chloride [172], and mercury [173]. Furthermore, some studies showed no significant increased risks of different pollutants with bladder cancer [174,175], leukemia [176,177], myeloma [178], brain cancer [5,79], breast cancer [63,113,179] (for more details, refer to Table S3).

The impact of different social factors on the cancer risks from pollution factors has also been studied. In a study [180], about 14% of census tracts in the St. Louis metropolitan area showed significantly increased cancer risk from air toxics, primarily in areas with high poverty, unemployment, and low education levels. Tracts with both high black racial isolation and economic isolation were found to be more than five times likely to be in toxic hotspots [180]. Likewise, another study in Memphis, Tennessee, geographically weighted regression analysis showed that the population living in the predominantly African American census tracts faced 6% higher cancer risk burden from air toxics than those in white concentrated tracts [181]. Furthermore, a study published in 2024 found that the higher proportions of black residents in suburban and rural areas were found to be significantly associated with increased estimated air toxic cancer risks, and similar positive associations were observed for Asian and Hispanic populations in nonurban areas in the US [182]. Also, the residents in Arab enclaves were found to have higher cancer risks from hazardous air pollution in the USA [183].

#### 3.2.2. Water Pollution and Land Pollution

Among the reviewed publications, 11 studies (summarized in Table S4) were found to study the association of different cancers with exposure to pollutants via water. Among them, eight studies [184,185,186,187,188,189,190,191] were related to heavy metals. The heavy metal exposure via water has been consistently linked to cancer risks. In Punjab, India [184], the excess lifetime cancer risks from arsenic-contaminated groundwater were way above the recommended USEPA threshold of 1 × 10^−4^. In Ghana, the carcinogenic risks from lead were found to be within the limits, whereas those from cadmium exceeded the USEPA limits [185]. Nickel, cadmium, and chromium in the groundwater near a landfill site in Thailand were found to have carcinogenic risks exceeding the USEPA limits [186]. In another study, the contamination of nickel, cadmium, and lead was higher than the USEPA limit in the Kilimambogo region of Kenya [191]. Furthermore, significant cancer risks of kidney cancer [187], prostate cancer [188], and bladder cancer [189] from arsenic exposure were found in Texas, Iowa, and Taiwan, respectively. Additionally, high environmental exposure to pentachlorophenol (PCP) was linked to significantly increased cancer incidences across multiple organ systems, including lymph, blood, nasopharynx, liver, gallbladder, and respiratory systems, among community populations along the Yangtze River in China [192]. In a study [193], the cancer risk associated with the consumption of drinking water contaminated with PFAs in Ronneby municipality in Sweden was examined. However, no significant associations were discovered. In addition, an article was found to be studying the cancer risks from radon exposure via water in a university area in Nigeria; however, the cancer risks were found to be within the acceptable thresholds [194].

Furthermore, only one study was found to be discussing the carcinogenic risk from land pollution among the reviewed publications [195]. The study discovered that the consumption of crops grown from the contaminated soils in the upper Crocodile River catchment in South Africa led to increased carcinogenic risks. The major contaminants were aluminum, iron, manganese, chromium, nickel, zinc, copper, and lead. Furthermore, a study found that Bisphenol A exposure significantly increased the risk of prostate cancer in Hong Kong Chinese men aged below 70 years [196].

### 3.3. Quality Appraisal Result

Based on the MMAT classification, the studies were of two types, Quantitative non-randomized (*n* = 126) and Quantitative descriptive (*n* = 42). The majority of the studies reviewed can be considered high-quality papers except for a few as they generally met the MMAT checklist criteria; 54 Quantitative non-randomized and 7 Quantitative description studies met all the five criteria and 59 Quantitative non-randomized studies and 32 Quantitative descriptive studies met four of the five MMAT criteria representing high-quality studies. However, among the 168 studies, three Quantitative non-randomized studies and two quantitative descriptive studies met only two criteria of the appraisal questions. Furthermore, among the Quantitative non-randomized studies, 45 studies failed the MMAT 3.1, “Are the participants representative of the target population?”, and among the Quantitative descriptive studies, 33 studies failed the question MMAT 4.5, “Is the statistical analysis appropriate to answer the research question?”

### 3.4. Meta-Analyses

**Figure 5:**
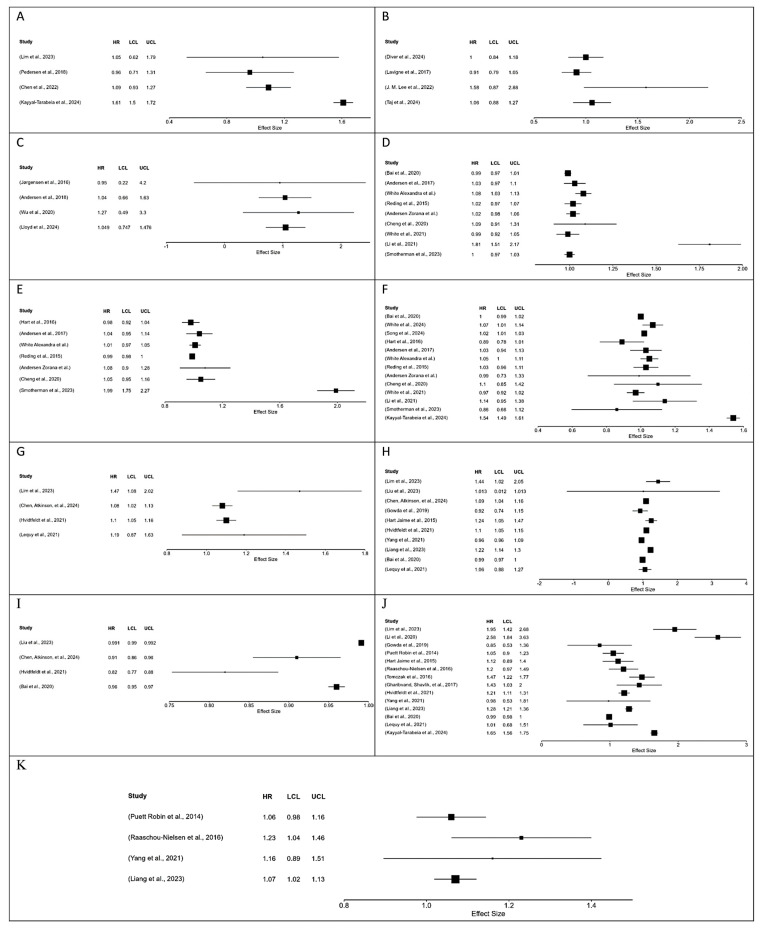
Forest plot showing the meta-analyses’ results. (**A**) Forest plot of included studies with HR of bladder cancer for PM_2.5_ exposure [36,49,174,175], (**B**) Forest plot of included studies with HR of blood cancer (leukemia) for PM_2.5_ exposure [34,72,80,120], (**C**) Forest plot of included studies with HR of brain cancer (Malignant) for PM_2.5_ exposure [5,75,77,79], (**D**) Forest plot of included studies with HR of breast cancer for NO_2_ exposure [58,59,60,61,62,63,64,67,70,113,117], (**E**) Forest plot of included studies with HR of breast cancer for PM_10_ exposure [58,61,62,63,64,65,70,113], (**F**) Forest plot of included studies with HR of breast cancer for PM_2.5_ exposure [33,36,58,59,60,61,62,63,64,65,66,67,69,70,113], (**G**) Forest plot of included studies with HR of lung cancer for black carbon exposure [39,44,49,71], (**H**) Forest plot of included studies with HR of lung cancer for NO_2_ exposure [37,39,42,44,46,49,57,67,71,106,111], (**I**) Forest plot of included studies with HR of lung cancer for ozone exposure [39,44,46,67,111], (**J**) Forest plot of included studies with HR of lung cancer for PM_2.5_ exposure [36,37,39,40,41,42,48,49,50,57,67,71,106,107,108,111], (**K**) Forest plot of included studies with HR of lung cancer for PM_10_ exposure [37,106,107,108,111].

A total of 11 meta-analyses were conducted in this study to evaluate the association of different pollutants with cancers, using the HR from multiple studies. Meta-analyses were predominantly feasible for only a few air pollutants due to limited comparable data for water and land pollution. The pollutants included are PM_2.5_, PM_10_, NO_2_, O_3_, and black carbon, and the data extracted from the studies and used in the meta-analyses are presented in Table S5. The results obtained from the meta-analyses are presented in Table 2 below, and the forest plots for the meta-analysis are shown in Figure 5.

Lung cancer was found to have a significant pooled HR with PM_2.5_, ozone, and black carbon. PM_2.5_ and black carbon had pooled HR of greater than 1, showing increased lung cancer risks with increased exposure to these air pollutants. However, exposure to ozone was inversely associated with lung cancer risk (HR = 0.941; 95% CI: 0.908–0.975). Similarly, breast cancer was significantly associated with NO_2_ with an HR of greater than 1. This shows the increased breast cancer with increased exposure to NO_2_. The I^2^ value of the 7 meta-analyses was found to be greater than 90%, showing very high heterogeneity. Moderate heterogeneity with I^2^ of 45.6% and *τ*^2^ of 0.008 was observed for the meta-analysis conducted for blood cancer (Leukemia) and PM_2.5_ exposure. However, very low heterogeneity was observed for the meta-analyses for lung cancer with PM_10_ (I^2^ = 19.33%, *τ*^2^ = 0.006), and black carbon (I^2^ = 0.059%, *τ*^2^ = 0.00001), and a zero I^2^ and *τ*^2^ value for malignant brain cancer with PM_2.5_ exposure.

## 4. Discussion

This review shows that addressing environmental pollution remains a crucial aspect in reducing the global cancer burden, as multiple associations have been observed between different pollutants and cancer risks across various organ systems in the human body. Particulate matter stands out with consistent and significant associations to cancers of different human organs, including the lung, breast, and brain, while ozone shows inconsistent yet significant associations that call for further exploration.

The population facing the greater impact from pollution-related problems is from low- and middle-income countries [197]; however, it was found that the major focus of the literature is on higher-income countries. Additionally, socio-demographic disparities are found to amplify the unequal exposure to environmental hazards, further deepening the cancer disparity. This suggests that future research and policies across the globe could benefit from incorporating these disparities in lessening the cancer burden.

Furthermore, substantial heterogeneity was observed (I^2^ > 90%) in the conducted meta-analyses. It suggests that the association between pollution and cancer is influenced by regional and methodological factors. For instance, the impact of PM_2.5_ may differ between industrial urban centers in Asia and lower-exposure environments in Europe due to differences in particle composition and population vulnerability. Moreover, variations in how studies controlled for confounding factors like socioeconomic status and lifestyle likely contribute to the observed variance in risk estimates.

### 4.1. Air Pollution and Its Association with Cancer Risks Across Multiple System

This review and meta-analysis showed the association of air pollutants with cancer across multiple systems, like the circulatory system, breast, central nervous system, and many more, despite the presence of some statistical inconsistencies across the studies. Similar findings were also reported in a previous review [198]. Several studies present a significant positive association of increased breast cancer risk with increased PM_2.5_ exposure [33,36,66]. However, statistically non-significant associations of breast cancer risk with PM_2.5_ exposure were also found in other studies [58,59,61,63]. Similarly, a significantly increased risk of leukemia with NO_2_ exposure was reported in a Danish cohort [34], but statistically non-significant associations were also discovered for the same [80,120]. However, the studies have suggested increased cancer risks across multiple systems from air pollution exposure. In a review done to assess the association of breast cancer risk with air pollution, a higher breast cancer risk was found to be associated with nitrogen oxides [199]. Similarly, in a Finnish cohort, increased exposure to engine exhaust was found to increase ovarian cancer, bladder cancer, and kidney cancer [200]. Furthermore, increased risk of bladder cancer was confirmed for occupational exposure to air pollutants like polycyclic aromatic hydrocarbons (PAHs). These studies and findings present the necessity of the call for greater global efforts towards air pollution control to reduce cancer incidences associated with it. To help countries across the globe in reducing air pollution effects on their citizens’ health, the World Health Organization (WHO) has been publishing air quality guidelines since 1987 [201]. Likewise, there are other multiple plan policies like for Europe; there is the ambient air quality and cleaner air directive by the European Union (EU) [202], the Industrial and livestock rearing emissions directive by the EU [203], the clean air act by the US [204], the Air Pollution Prevention and Control Action Plan by China, [205], etc., in different countries to improve the air quality. Governments can follow these guidelines to improve the air quality in their country. Stringent exhaust regulations and incentives to those who adhere to the regulations set can be a way to improve air quality.

### 4.2. Ozone Exposure and Its Inconsistent Association with Cancer Risks

Interestingly, in this review, it was seen that ozone exposure was inversely related to cancer risks, lung cancer [39,44,46,67], liver cancer [83], and meningioma [81], in multiple studies, showing reduced lung cancer risks with increased ozone exposure. It is also evident from the findings of our meta-analysis. The inverse association can result from the co-pollutant confounding. It has been discovered that in high-traffic urban areas, nitric oxide emissions react and lead to lower concentration of ozone in locations where other carcinogens, such as PM_2.5_ and NO_2_, are at their highest [206]. However, ozone is a highly potent oxidant, and oxidative stress is one of the established pathways for carcinogenesis [14]. One of the studies reported an increased risk of lung cancer with ozone exposure [43]. This shows inconsistent findings in the studies between ozone exposure and cancer risks. Furthermore, ozone exposure is related to multiple human health impacts and is considered a harmful pollutant [207,208,209]. There were 432,100 deaths attributable to ozone globally in 2019 [210]. This depicts the potential requirement of stringent actions for controlling air pollution from ozone. Currently, multiple acts and policies are in place to facilitate the control of air pollution from ozone. In the US, the Environmental Protection Agency (EPA) sets standards for ground-level ozone concentration [211], ambient air quality, and the Cleaner Air Directive for Europe by the EU has set standards for European countries [202], and many more. However, the limited volume of literature discussing the association of ozone exposure with cancer risks, with inconsistent and limited associations, calls for further comprehensive research.

### 4.3. Particulate Matter Exposure and Its Strong Association with Cancer Risks

In this review, it was discovered that among the different air pollutants, particulate matters (PM_2.5_, PM_10_, PM_2.5_, UFPs) are the ones that are most studied for cancer risks and are also greatly linked to multiple cancers, including but not limited to lung cancer, brain cancer, and breast cancer. The small particles, when inhaled, can be easily absorbed into the blood during respiration and can travel and get deposited anywhere throughout the body [212]. This greatly increases the concern that particulate matter may exert carcinogenic effects in almost any part of the human body. Multiple reviews and studies have found associations of particulate matter with different cancers, lung [37,40,45,213], bladder cancer [36,213], gastrointestinal cancer [89,214], and many more. These findings emphasize that prioritizing the control of particulate matter exposure could be relevant public health strategy to reduce cancer risks. If given priority, striving to adhere to the set guidelines by the WHO, EU, and other bodies, alongside strong monitoring and implementing effective measures to reduce the induction of particulate matter to the ambient atmosphere, could help in reducing the cancer burden related to them.

### 4.4. Disparities in Research Focus Based on Pollution Type

It was observed that among the reviewed studies, greater focus was on air pollution, and very few studies dealt with other (land and water) pollution and cancer risks. This shows a disparity in research on pollution-related cancer risks based on the type of pollution. However, even with such a low number of studies (when compared to the number of studies focused on air pollution) reviewed, the waterborne pollutants, specifically heavy metals like lead, arsenic, cadmium, etc., were found to be increasing the cancer risks. Several studies reported carcinogenic risks from such water pollutants exceeding the USEPA’s acceptable thresholds in countries like India, Thailand, and Ghana [184,185,186]. Even in the US, increased cancer risks were associated with exposure to waterborne pollutants [187,188]. Such levels of pollutants in water sources leading to increased cancer risks suggest either there might be a lack of stringent laws and regulations, or the implementation and monitoring parts of the existing law might be lacking. Various standards and guidelines are prevalent across the globe, like guidelines from the WHO to limit the levels of heavy metals [215]. Likewise, there are the Clean Water Act [216] and the Safe Drinking Water Act [217] in place to control and regulate water in the USA. Europe is guided by the EU Drinking Water Directives [218], and similar acts are present in countries like China, India, and others. With the presence of statutes, the problem might be in their strict implementation. This suggests that stronger implementation plans for the existing guidelines may be a key consideration for policymakers across the globe.

### 4.5. Socioeconomic and Socio-Demographic Disparities in Cancer Burden

This review also presented the prevalence of socioeconomic and socio-demographic disparities that are related to increased cancer risks. Several studies have shown the disproportionately greater burden of cancer risks by race, ethnicity, socioeconomic status, and urbanicity [180,181,182]. People who live in poor and racially segregated areas of cities or industrial regions were more exposed to pollution and were more susceptible to air toxics and particles [96]. This type of socioeconomic inequality was spread both among adults and children [149]. These disparities demonstrate an urgent need for interventions to address inequality in exposure to the environment and access to healthcare. Policymakers should consider such socioeconomic and socio-demographic disparities while making policies. The unequal incidence of cancer among different groups can be minimized with the help of environmentally fair policies and specific measures.

### 4.6. Uneven Global Focus on Pollution-Related Cancer Research

This review saw that a major portion of the studies (almost 79%) is from just six economically strong countries: the USA, China, Denmark, Canada, Taiwan, and South Korea. This shows an uneven global focus of pollution-related cancer research, with disproportionate focus on the population of relatively wealthy and developed nations. Furthermore, except for China, these countries maintain good air quality [219], raising concerns about whether the findings from these studies adequately capture the health effects in regions with more severe pollution impacts. The greatest economic burden, along with 92% of pollution-related deaths, occurs in low- and middle-income countries (LMICs) [197]. According to the WHO, the premature deaths due to air pollution alone in LMICs are about 3.68 million each year, which is about eight times the mortality rate in high-income countries [3]. Furthermore, cancer burden appears to be increasing in developing countries [197]. However, very little has been done to overcome the pollution-related challenges in those countries [1]. To better assess the global carcinogenic impacts, research focus should also be directed to the population in countries and places where there is a higher impact of pollution and poor regulatory systems. This seems to be lacking in the current literature. Increased funding and collaborative research initiatives from high-income countries can be a potential way out. Such actions could help generate more representative data, raise global awareness, and support targeted interventions where they are most needed.

### 4.7. Future Risks

In this review, it was seen that increased exposure to different air pollutants increased cancer risks across multiple parts of the human body. For this reason, it can be said that controlling environmental pollution is a plausible approach to address the global cancer burden. This requires proper awareness about pollution; however, the communication is still not adequate [220]. Furthermore, the current pollution emissions are being further challenged by some new trends in politics and industry. For instance, the withdrawal of US from the Paris Agreement [221], shifting of energy focus towards fossil fuels via executive order to declare national energy emergency [222], may disrupt worldwide efforts to cut greenhouse gas emissions and directly result in increased air pollutants’ emissions such as particulate matter, nitrogen oxides and black carbon, which are found to be associated with cancer risks as discovered in this review. Similarly, rising global tensions and wars have led to a significant rise in the amount of air toxics and particulates in the atmosphere [223]. Furthermore, the ongoing trade wars have led to a change in global logistics. This can lead to more emissions due to longer supply chains, further aggravating the pollution situation [224]. In addition, there is an ongoing global race to produce more battery electric vehicles in the name of reducing greenhouse gas emissions from vehicles using fossil fuels [225]. However, the lack of consideration for proper after-use management of harmful battery chemicals also increases the concern of pollution [226]. Furthermore, it is increasing the mining of metals like lithium and cadmium, which has further increased the risk of environmental contamination [227]. This suggests that a global understanding of the cumulative effort on pollution control can be a way forward to address the growing global cancer burden concern.

## 5. Limitation

First, the majority of the studies were observational studies (such as cohort and case–control designs). This means that even though the studies presented strong associations, the direct causality of exposures and cancer cannot be proven definitively. Next, in the environmental research, exposure measurement errors are a common challenge. In the majority of the included studies, fixed-site air monitoring stations have been used as a proxy for individual exposure. This may lead to some misclassification of personal risk. Furthermore, the studies reported different effect measures (HR, OR, and RR); this further added to the variation in observed results.

Another limitation of this study is the high statistical heterogeneity in the meta-analyses. While the findings indicate a consistent positive association between pollution and cancer risk, the high I^2^ values mean the exact magnitude of this risk should be interpreted with caution. This heterogeneity is likely driven by regional differences, variations in covariate adjustment, and the use of non-standardized exposure increments across the included studies. Future research with more standardized exposure metrics and larger datasets for subgroup analysis is required to further clarify these variations.

The potential for publication bias also exists, as studies with statistically significant positive results are more likely to be published than those showing no association. Finally, this review was restricted to studies published in English only after 2014. This leaves a vacuum in the literature, as significant research published in other languages, including Russian and Chinese, were excluded.

## 6. Conclusions

This review presented the increased cancer risks from different pollutants, highlighting the need to control their emission, be it air pollutants, water pollutants, or land pollutants. It was seen that research was greatly focused on air pollutants, and among them, the cancer risks of PM_2.5_ were the most studied. Furthermore, it showed significant associations with several cancer types. Also, the heavy metal contamination in water and land poses notable carcinogenic risks; however, these areas remain relatively less explored. Furthermore, more than two-thirds of the reviewed studies originated from a few high-income countries with relatively cleaner environments, demonstrating a global disparity in research focus that has been overlooking populations in LMICs with increasing pollution and cancer burdens. Additionally, this review showed that socioeconomic and socio-demographic inequalities, including racial, gender, education level, and urbanicity, have an impact on the cancer risks from pollution exposure. All in all, more comprehensive data collection and an increased research focus on underrepresented regions are essential for understanding the pollution-related cancer situation better and ultimately mitigating those cancer risks globally.

## Figures and Tables

**Table 1:** PECO search strategy for the systematic review.

Population	Exposure	Control	Outcome
General human population	Air pollution, water pollution, land pollution, pollution, hazardous waste, pollutants, PM, particulate matter, ozone, O_3_, diesel PM, lead, toxic waste, socio-demographic, socioeconomic.	Not specified	Cancer

**Table 2:** Meta-analyses results.

Cancer	Pollutant	Pooled Effect Size (HR [95%CI])	Standard Error	*τ*	*τ* ^2^	I^2^	*p*-Value
Bladder Cancer	PM_2.5_	1.2 [0.811, 1.589]	0.198	0.368	0.136	94.145	<0.001
Blood Cancer (leukemia)	PM_2.5_	1.013 [0.876, 1.15]	0.07	0.92	0.008	45.645	<0.001
Brain Cancer (malignant)	PM_2.5_	1.059 [0.802, 1.317]	0.131	0	0	0	<0.001
Breast Cancer	NO_2_	1.064 *** [1.011, 1.117]	0.027	0.071	0.005	91.068	<0.001
Breast Cancer	PM_10_	1.16 [0.823, 1.498]	0.138	0.359	0.129	99.465	<0.001
Breast Cancer	PM_2.5_	1.063 [0.98, 1.146]	0.042	0.138	0.019	98.283	<0.001
Lung Cancer	Black Carbon	1.096 *** [1.014, 1.179]	0.026	0.001	0.00001	0.059	<0.001
Lung Cancer	NO_2_	1.08 [0.996, 1.18]	0.041	0.101	0.01	91.195	<0.001
Lung Cancer	Ozone	0.941 [.908, 0.975]	0.017	0.029	0.0009	95.527	<0.001
Lung Cancer	PM_2.5_	1.347 *** [1.158, 1.536]	0.097	0.334	0.111	98.237	<0.001

*** *p*-value < 0.001 and CI not crossing 1.

## Data Availability

No new data were created or analyzed in this study. Data sharing is not applicable to this article.

## Supplementary Materials

The following supporting information can be downloaded at: https://www.mdpi.com/article/10.3390/ijerph23040429/s1; Table S1: PRISMA 2020 checklist; Table S2: Rejection reasons for articles in the different screening phases; Table S3: Review summary of the publications studying the association of air pollution with cancer; Table S4: Review summary of the publications studying the association of water and land pollution with cancer; Table S5: Data used for meta-analyses.
