# Supplementary material for: Impact of Pollution on Cancer: A Systematic Review and Meta-Analysis with Focus on Air Pollution
Source: Int J Environ Res Public Health. 2026 Mar 30;23(4):429. doi: 10.3390/ijerph23040429 (PMC13116831; doi:10.3390/ijerph23040429)
Supplement: Supplementary file 1 [file ijerph-23-00429-s001.zip › ijerph-4175881-supplementary.pdf]

PRISMA 2020 Checklist

Table S1: PRISMA 2020 Checklist

Section and Topic	Item #	Checklist item	Location where item is reported
**TITLE**			
Title	1	Identify the report as a systematic review.	1
**ABSTRACT**			
Abstract	2	See the PRISMA 2020 for Abstracts checklist.	1-2
**INTRODUCTION**			
Rationale	3	Describe the rationale for the review in the context of existing knowledge.	2-3
Objectives	4	Provide an explicit statement of the objective(s) or question(s) the review addresses.	2-3
**METHODS**			
Eligibility criteria	5	Specify the inclusion and exclusion criteria for the review and how studies were grouped for the syntheses.	4
Information sources	6	Specify all databases, registers, websites, organisations, reference lists and other sources searched or consulted to identify studies. Specify the date when each source was last searched or consulted.	3-4
Search strategy	7	Present the full search strategies for all databases, registers and websites, including any filters and limits used.	3-4
Selection process	8	Specify the methods used to decide whether a study met the inclusion criteria of the review, including how many reviewers screened each record and each report retrieved, whether they worked independently, and if applicable, details of automation tools used in the process.	4
Data collection process	9	Specify the methods used to collect data from reports, including how many reviewers collected data from each report, whether they worked independently, any processes for obtaining or confirming data from study investigators, and if applicable, details of automation tools used in the process.	4-5
Data items	10a	List and define all outcomes for which data were sought. Specify whether all results that were compatible with each outcome domain in each study were sought (e.g. for all measures, time points, analyses), and if not, the methods used to decide which results to collect.	4-5
	10b	List and define all other variables for which data were sought (e.g. participant and intervention characteristics, funding sources). Describe any assumptions made about any missing or unclear information.	4-5
Study risk of bias assessment	11	Specify the methods used to assess risk of bias in the included studies, including details of the tool(s) used, how many reviewers assessed each study and whether they worked independently, and if applicable, details of automation tools used in the process.	4
Effect measures	12	Specify for each outcome the effect measure(s) (e.g. risk ratio, mean difference) used in the synthesis or presentation of results.	5
Synthesis methods	13a	Describe the processes used to decide which studies were eligible for each synthesis (e.g. tabulating the study intervention characteristics and comparing against the planned groups for each synthesis (item #5)).	4-5
	13b	Describe any methods required to prepare the data for presentation or synthesis, such as handling of missing summary statistics, or data conversions.	4-5
	13c	Describe any methods used to tabulate or visually display results of individual studies and syntheses.	4-5
	13d	Describe any methods used to synthesize results and provide a rationale for the choice(s). If meta-analysis was performed, describe the model(s), method(s) to identify the presence and extent of statistical heterogeneity, and software package(s) used.	4-5
	13e	Describe any methods used to explore possible causes of heterogeneity among study results (e.g. subgroup analysis, meta-regression).	NA
	13f	Describe any sensitivity analyses conducted to assess robustness of the synthesized results.	NA
Reporting bias	14	Describe any methods used to assess risk of bias due to missing results in a synthesis (arising from reporting biases).	NA

Section and Topic	Item #	Checklist item	Location where item is reported
assessment			
Certainty assessment	15	Describe any methods used to assess certainty (or confidence) in the body of evidence for an outcome.	NA
**RESULTS**			
Study selection	16a	Describe the results of the search and selection process, from the number of records identified in the search to the number of studies included in the review, ideally using a flow diagram.	5-7
	16b	Cite studies that might appear to meet the inclusion criteria, but which were excluded, and explain why they were excluded.	NA
Study characteristics	17	Cite each included study and present its characteristics.	Table S3 and S4
Risk of bias in studies	18	Present assessments of risk of bias for each included study.	NA
Results of individual studies	19	For all outcomes, present, for each study: (a) summary statistics for each group (where appropriate) and (b) an effect estimate and its precision (e.g. confidence/credible interval), ideally using structured tables or plots.	Table S3 and S4
Results of syntheses	20a	For each synthesis, briefly summarise the characteristics and risk of bias among contributing studies.	NA
	20b	Present results of all statistical syntheses conducted. If meta-analysis was done, present for each the summary estimate and its precision (e.g. confidence/credible interval) and measures of statistical heterogeneity. If comparing groups, describe the direction of the effect.	13-15
	20c	Present results of all investigations of possible causes of heterogeneity among study results.	NA
	20d	Present results of all sensitivity analyses conducted to assess the robustness of the synthesized results.	NA
Reporting biases	21	Present assessments of risk of bias due to missing results (arising from reporting biases) for each synthesis assessed.	NA
Certainty of evidence	22	Present assessments of certainty (or confidence) in the body of evidence for each outcome assessed.	NA
**DISCUSSION**			
Discussion	23a	Provide a general interpretation of the results in the context of other evidence.	15-18
	23b	Discuss any limitations of the evidence included in the review.	18,19
	23c	Discuss any limitations of the review processes used.	18,19
	23d	Discuss implications of the results for practice, policy, and future research.	15-18
**OTHER INFORMATION**			
Registration and protocol	24a	Provide registration information for the review, including register name and registration number, or state that the review was not registered.	3
	24b	Indicate where the review protocol can be accessed, or state that a protocol was not prepared.	3
	24c	Describe and explain any amendments to information provided at registration or in the protocol.	NA
Support	25	Describe sources of financial or non-financial support for the review, and the role of the funders or sponsors in the review.	19
Competing interests	26	Declare any competing interests of review authors.	19
Availability of data, code and other materials	27	Report which of the following are publicly available and where they can be found: template data collection forms; data extracted from included studies; data used for all analyses; analytic code; any other materials used in the review.	12, TableS5

Table S2. Rejection reasons for articles in the different screening phases.

Screening phase	Title of the study	Reason for rejection
Title Screening	"All excluded studies"	Not at all relevant.
Abstract screening	Multidimensional structural racism and estimated cancer risk from traffic-related air pollution	The independent variable is out of the scope of the study.
	Effect of residential air cleaning interventions on risk of cancer associated with indoor semi-volatile organic compounds: a comprehensive simulation study	The independent variable is out of the scope of the study.
	Residential proximity to green spaces and breast cancer risk: The multicase-control study in Spain (MCC-Spain)	The independent variable is out of the scope of the study.
	Parental occupational exposure to benzene and the risk of childhood cancer: A census-based cohort study	The independent variable is out of the scope of the study.
	Cancer risks from exposure to vehicular air pollution: a household level analysis of intra-ethnic heterogeneity in Miami, Florida	The independent variable is out of the scope of the study.
	Exposure to ionizing radiation and brain cancer incidence: The Life Span Study cohort	The independent variable is out of the scope of the study.
	Occupational exposure to pesticides and bladder cancer risk	The independent variable is out of the scope of the study.
	Residential Exposure to Road and Railway Noise and Risk of Prostate Cancer: A Prospective Cohort Study	The independent variable is out of the scope of the study.
	Exposure to polychlorinated biphenyls and hexachlorobenzene, semen quality and testicular cancer risk	The independent variable is out of the scope of the study.
	Residential Exposure to Estrogen Disrupting Hazardous Air Pollutants and Breast Cancer Risk The California Teachers Study	The independent variable is out of the scope of the study.
	Vegetarian Dietary Patterns and the Risk of Colorectal Cancers	The independent variable is out of the scope of the study.
	Cancer risk of incremental exposure to polycyclic aromatic hydrocarbons in electrocautery smoke for mastectomy personnel	The independent variable is out of the scope of the study.
	Long-Term Effect of Temperature Increase on Liver Cancer in Australia: A Bayesian Spatial Analysis	The independent variable is out of the scope of the study.
	Pesticide exposure and lung cancer risk: A case-control study in Nakhon Sawan, Thailand	The independent variable is out of the scope of the study.
	Cancer risk assessment for occupational exposure to chromium and nickel in welding fumes from pipeline construction, pressure container manufacturing, and shipyard building in Taiwan	The independent variable is out of the scope of the study.
	Risk factors for central nervous system tumors in children: New findings from a casecontrol study	The independent variable is out of the scope of the study.
	Household inhalants exposure and nasopharyngeal carcinoma risk: A large-scale case-control study in Guangdong, China	The independent variable is out of the scope of the study.
	Ecological association between residential natural background radiation exposure and the incidence rate of childhood central nervous system tumors in France, 2000-2012	The independent variable is out of the scope of the study.
	Parental occupational exposure to pesticides, animals and organic dust and risk of childhood leukemia and central nervous system tumors: Findings from the International Childhood Cancer Cohort Consortium (I4C)	The independent variable is out of the scope of the study.
	Geographical variation in lung cancer risk associated with road traffics in Jiading District, Shanghai	The independent variable is out of the scope of the study.
	Exposure to hazardous air pollutants and risk of incident breast cancer in the nurses' health study II	The independent variable is out of the scope of the study.
	Residential proximity to environmental pollution sources and risk of rare tumors in children	The independent variable is out of the scope of the study.

	Household air pollution and risk of incident lung cancer in urban China: A prospective cohort study	The independent variable is out of the scope of the study.
	Lung Cancer Risk from Radon in Marcellus Shale Gas in Northeast U.S. Homes	The independent variable is out of the scope of the study.
	Residence in Proximity of an Iron Foundry and Risk of Lung Cancer in the Municipality of Trieste, Italy, 1995-2009	The independent variable is out of the scope of the study.
	Childhood leukemia and residential proximity to industrial and urban sites	The independent variable is out of the scope of the study.
	Diesel engine exhaust and lung cancer mortality: time-related factors in exposure and risk	The independent variable is out of the scope of the study.
	Doses and lung cancer risks from exposure to radon and plutonium	The independent variable is out of the scope of the study.
	Occupational brain cancer risks in Umbria (Italy), with a particular focus on steel foundry workers	The independent variable is out of the scope of the study.
	Inhalation and dermal exposure to atmospheric polycyclic aromatic hydrocarbons and associated carcinogenic risks in a relatively small city	The independent variable is out of the scope of the study.
	Risk factors for lung cancer in the Pakistani population	The independent variable is out of the scope of the study.
	Cancer Incidence Relation to Heavy Metals in Soils of Kyzylorda Region of Kazakhstan	The independent variable is out of the scope of the study.
	Association between Airport Ultrafine Particles and Lung Cancer Risk: The Multiethnic Cohort Study	The independent variable is out of the scope of the study.
	Simultaneous measurements of radon, thoron and thoron progeny and induced cancer risk assessment in Djeno, Pointe-Noire, Republic of Congo	The independent variable is out of the scope of the study.
	Breast cancer risk for the joint exposure to metals and metalloids in women: Results from the EPIC-Spain cohort	The independent variable is out of the scope of the study.
	Long-term exposure to several constituents and sources of PM(2.5) is associated with incidence of upper aerodigestive tract cancers but not gastric cancer: Results from the large pooled European cohort of the ELAPSE project	The independent variable is out of the scope of the study.
	Household air pollution and risk of incident lung cancer in urban China: A prospective cohort study	The independent variable is out of the scope of the study.
	Lung cancer risk and exposure to air pollution: a multicenter North China case-control study involving 14604 subjects	The independent variable is out of the scope of the study.
	Occupational risk variation of nasopharyngeal cancer in the Nordic countries	The independent variable is out of the scope of the study.
	Low-Level Environmental Mercury Exposure and Thyroid Cancer Risk Among Residents Living Near National Industrial Complexes in South Korea: A Population-Based Cohort Study	The independent variable is out of the scope of the study.
	Trends of Esophageal Cancer Incidence and Mortality and Its Influencing Factors in China	The independent variable is out of the scope of the study.
	Incidence and mortality trends of thyroid cancer from 1980 to 2016	The independent variable is out of the scope of the study.
	Light pollution as a factor in breast and prostate cancer	The independent variable is out of the scope of the study.
	Bisphenol-A exposure and risk of breast and prostate cancer in the Spanish European Prospective Investigation into Cancer and Nutrition study	The independent variable is out of the scope of the study.
	Commute patterns, residential traffic-related air pollution, and lung cancer risk in the prospective UK Biobank cohort study	The independent variable is out of the scope of the study.
	An Examination of National Cancer Risk Based on Monitored Hazardous Air Pollutants	The independent variable is out of the scope of the study.

	Impact of environmental exposure to persistent organic pollutants on lung cancer risk	The independent variable is out of the scope of the study.
	Dioxin exposure and breast cancer risk in a prospective cohort study	The independent variable is out of the scope of the study.
	Carcinogenic risk from exposure to PM(2.5) bound polycyclic aromatic hydrocarbons in rural settings	The independent variable is out of the scope of the study.
	Chronic long-term exposure to cadmium air pollution and breast cancer risk in the French E3N cohort	The independent variable is out of the scope of the study.
	Carcinogenic and non-carcinogenic risk assessment of heavy metals in PM2.5 air pollutant in Ulaanbaatar, Mongolia during the wintertime	No cancer outcome reported.
	Heavy Metals Pollution and Potential Health Risks: The Case of the Koche River, Tatek Industrial Zone, Burayu, Ethiopia	No cancer outcome reported.
	Long-term exposure to ambient air pollution and risk of lung cancer - A comparative analysis of incidence and mortality in four administrative cohorts in the ELAPSE study	No cancer outcome reported.
	Association of Age with Non-muscle-invasive Bladder Cancer: Unearthing a Biological Basis for Epidemiological Disparities?	No cancer outcome reported.
	How long-term PM exposure may affect all-site cancer mortality: Evidence from a large cohort in southern China	No cancer outcome reported.
	Evaluating Social Determinants of Health Related to Cancer Survivorship and Quality of Care	No cancer outcome reported.
	Association between long-term exposure to air pollution and the risk of incident laryngeal cancer: a longitudinal UK Biobank-based study	Not an empirical study with cancer outcome reported.
	Impact of social and economic factors on global thyroid cancer incidence and mortality	Not an empirical study with cancer outcome reported.
	Methylated polycyclic aromatic hydrocarbons from household coal use across the life course and risk of lung cancer in a large cohort of 42,420 subjects in Xuanwei, China	Not an empirical study with cancer outcome reported.
	Carcinogenic and non-carcinogenic health risk assessment of air pollutant in a steel industry	Not an empirical study with cancer outcome reported.
	Carcinogenic and Non-carcinogenic Health Risk Assessment of Heavy Metals in Njaba River, Imo State, Nigeria	Not an empirical study with cancer outcome reported.
	Carcinogenic and Non-carcinogenic Health Risk Assessment of Heavy Metals in Ground Drinking Water Wells of Bandar Abbas	Not an empirical study with cancer outcome reported.
	Evaluation of Role of Heavy Metals in Causation of Colorectal Cancer	Not an empirical study with cancer outcome reported.
	Heavy metals exposure, carcinogenic and non-carcinogenic human health risks assessment of groundwater around mines in Joghatai, Iran	Not an empirical study with cancer outcome reported.
	Non-carcinogenic and Carcinogenic Risk Assessment of Trace Elements of PM2.5 During Winter and Pre-monsoon Seasons in Delhi: A Case Study	Not an empirical study with cancer outcome reported.
	Carcinogenic and non-carcinogenic health risks of metal(oid)s in tap water from Ilam city, Iran	Not an empirical study with cancer outcome reported.
	Air pollution and environmental injustice: Are the socially deprived exposed to more PM2.5 pollution in Hong Kong?	Not an empirical study with cancer outcome reported.
	Carcinogenic and Non-carcinogenic Risk Assessment of Metals in Groundwater via Ingestion and Dermal Absorption Pathways for Children and Adults in Malwa Region of Punjab	Not an empirical study with cancer outcome reported.
	Cancer incidence in urban, rural, and densely populated districts close to core cities in Bavaria, Germany	Not an empirical study with cancer outcome reported.
	Assessment of Carcinogenic and Non-Carcinogenic Risk from Exposure to Uranium in Groundwater from Western Haryana, India	Not an empirical study with cancer outcome reported.
	Atmospheric fine particulate matter and breast cancer mortality: a population-based cohort study	Not an empirical study with cancer outcome reported.

	Smoking status, usual adult occupation, and risk of recurrent urothelial bladder carcinoma: data from The Cancer Genome Atlas (TCGA) Project	Not an empirical study with cancer outcome reported.
	Lung cancer risk by polycyclic aromatic hydrocarbons in a Mediterranean industrialized area	Not an empirical study with cancer outcome reported.
	Lung cancer and urbanization level in a region of Southern Europe: influence of socio-economic and environmental factors	Not an empirical study with cancer outcome reported.
	Cancer Mortality Risks from Long-term Exposure to Ambient Fine Particle	Not an empirical study with cancer outcome reported.
	Central nervous system (CNS) cancer in children and young people in the European Union and its involvements with socio-economic and environmental factors	Not an empirical study with cancer outcome reported.
	Household-level disparities in cancer risks from vehicular air pollution in Miami	Not an empirical study with cancer outcome reported.
	Socio-economic and cultural vulnerabilities to cervical cancer and challenges faced by patients attending care at Tikur Anbessa Hospital: a cross sectional and qualitative study	Not an empirical study with cancer outcome reported.
	Racial disparities in squamous cell carcinoma of the oral tongue among women: A SEER data analysis	Not an empirical study with cancer outcome reported.
	Traffic Exposure and Breast Cancer Mortality by Area of Residence: Incorporating Clinical and Socioeconomic Data	Not an empirical study with cancer outcome reported.
	Association between urban benzene pollution and incidence of acute myeloid leukemia	Not an empirical study with cancer outcome reported.
	Asbestos exposure and the mesothelioma incidence in Poland	Not an empirical study with cancer outcome reported.
	Environmental and economic factors of regional variation in childhood cancer across Russia	Not an empirical study with cancer outcome reported.
	Cadmium exposure and the risk of breast cancer in Chaoshan population of southeast China	Not an empirical study with cancer outcome reported.
	Malignant mesothelioma due to non-occupational asbestos exposure from the Italian national surveillance system (ReNaM): Epidemiology and public health issues	Not an empirical study with cancer outcome reported.
	Correlation of breast cancer incidence with the number of motor vehicles and consumption of gasoline in Korea	Not an empirical study with cancer outcome reported.
	Thyroid Cancer Incidence Rates in North Dakota are Associated with Land and Water Use	Not an empirical study with cancer outcome reported.
	Exposure of metals and PAH through local foods and risk of cancer in a historically contaminated glassworks area	Not an empirical study with cancer outcome reported.
	A geographically weighted regression approach to investigate air pollution effect on lung cancer: A case study in Portugal	Not an empirical study with cancer outcome reported.
	Estimates of the current and future burden of lung cancer attributable to PM(2.5) in Canada	Not an empirical study with cancer outcome reported.
	Wildland firefighter smoke exposure and risk of lung cancer and cardiovascular disease mortality	Not an empirical study with cancer outcome reported.
	Baseline heavy metals in plant species from some industrial and rural areas: Carcinogenic and non-carcinogenic risk assessment	Not an empirical study with cancer outcome reported.
	Lung cancer risk assessment due to traffic-generated particles exposure in urban street canyons: A numerical modelling approach	Not an empirical study with cancer outcome reported.
	The association between heavy metal soil pollution and stomach cancer: a case study in Hangzhou City, China	Not an empirical study with cancer outcome reported.
	Chemical partitioning of fine particle-bound As, Cd, Cr, Ni, Co, Pb and assessment of associated cancer risk due to inhalation, ingestion and dermal exposure	Not an empirical study with cancer outcome reported.

	Correlation Analysis of PM(10) and the Incidence of Lung Cancer in Nanchang, China	Not an empirical study with cancer outcome reported.
	Hazardous air pollutants and primary liver cancer in Texas	Not an empirical study with cancer outcome reported.
	Female lung cancer mortality and long-term exposure to particulate matter in Italy	Not an empirical study with cancer outcome reported.
	Atmospheric fine particulate matter and breast cancer mortality: a population-based cohort study	Not an empirical study with cancer outcome reported.
	Residential cancer cluster investigation nearby a Superfund Study Area with trichloroethylene contamination	Not an empirical study with cancer outcome reported.
	Carcinogenic Air Toxics Exposure and Their Cancer-Related Health Impacts in the United States	Not an empirical study with cancer outcome reported.
	Evaluating Evidence for Association of Human Bladder Cancer with Drinking-Water Chlorination Disinfection By-Products	Not an empirical study with cancer outcome reported.
	Relationship between arsenic-containing drinking water and skin cancers in the arseniasis endemic areas in Taiwan	Not an empirical study with cancer outcome reported.
	Calculation of lifetime lung cancer risks associated with radon exposure, based on various models and exposure scenarios	Not an empirical study with cancer outcome reported.
	Risk of leukaemia or cancer in the central nervous system among children living in an area with high indoor radon concentrations: results from a cohort study in Norway	Not an empirical study with cancer outcome reported.
	Personal and indoor PM2.5 exposure from burning solid fuels in vented and unvented stoves in a rural region of China with a high incidence of lung cancer	Not an empirical study with cancer outcome reported.
	The association between ambient PM(2.5)'s constituents exposure and cervical cancer survival	Not an empirical study with cancer outcome reported.
	Trends in childhood leukemia incidence in urban countries and their relation to environmental factors, including space weather	Not an empirical study with cancer outcome reported.
	Evaluating county-level lung cancer incidence from environmental radiation exposure, PM(2.5), and other exposures with regression and machine learning models	Not an empirical study with cancer outcome reported.
	Exploring the association of PM(2.5) with lung cancer incidence under different climate zones and socioeconomic conditions from 2006 to 2016 in China	Not an empirical study with cancer outcome reported.
	Disease Burden and Prediction Analysis of Tracheal, Bronchus, and Lung Cancer Attributable to Residential Radon, Solid Fuels, and Particulate Matter Pollution Under Different Sociodemographic Transitions From 1990 to 2030	Not an empirical study with cancer outcome reported.
	Methylated polycyclic aromatic hydrocarbons from household coal use across the life course and risk of lung cancer in a large cohort of 42,420 subjects in Xuanwei, China	Not an empirical study with cancer outcome reported.
	INDOOR RADON CONCENTRATION AND EXCESS LIFETIME CANCER RISK	Not an empirical study with cancer outcome reported.
	Lack of knowledge is the leading key for the growing cervical cancer incidents in Bangladesh: A population based, cross-sectional study	Not an empirical study with cancer outcome reported.
	A Mixed Methods Population Health Approach to Explore Radon-Induced Lung Cancer Risk Perception in Canada	Not an empirical study with cancer outcome reported.
	Environmental asbestos exposure and clustering of malignant mesothelioma in community: a spatial analysis in a population-based case-control study	Not an empirical study with cancer outcome reported.
	Regional interaction of lung cancer incidence influenced by PM(2.5) in China	Not an empirical study with cancer outcome reported.
	Correction to: Association between cancer risk and polycyclic aromatic hydrocarbons' exposure in the ambient air of Ahvaz, southwest of Iran	Not an empirical study with cancer outcome reported.
	The association between air pollution level and breast cancer risk in Taiwan	Not an empirical study with cancer outcome reported.

	Lung cancer mortality attributable to residential radon exposure in Spain and its regions	Not an empirical study with cancer outcome reported.
	The epidemiological trends in the burden of lung cancer attributable to PM(2.5) exposure in China	Not an empirical study with cancer outcome reported.
	Childhood Leukemia in Small Geographical Areas and Proximity to Industrial Sources of Air Pollutants in Three Colombian Cities	Not an empirical study with cancer outcome reported.
	Evaluation of radon exposure risk and lung cancer incidence/mortality in South-eastern Italy	Not an empirical study with cancer outcome reported.
	Assessment of arsenic exposure by drinking well water and associated carcinogenic risk in peri-urban areas of Vehari, Pakistan	Not an empirical study with cancer outcome reported.
	Long-term Exposure to PM10 Increases Lung Cancer Risks: A Cohort Analysis	No cancer outcome reported.
	Socio-demographic determinants of cervical cancer screening uptake among women of child-bearing age in Mangochi, Malawi: a facility-based cross-sectional study	No cancer outcome reported.
	Socio-Demographic Disparities in Gastric Adenocarcinoma: A Population-Based Study	No cancer outcome reported.
	Educational level, management and outcomes in small-cell lung cancer (SCLC): A population-based cohort study	No cancer outcome reported.
	IS POVERTY ANOTHER CAUSE OF CANCER? AN EMPIRICAL ANALYSIS	No cancer outcome reported.
	Long-term exposure to urban air pollution and lung cancer mortality: A 12-year cohort study in Northern China	No cancer outcome reported.
	Long-term exposure to traffic noise and risk of incident colon cancer: A pooled study of eleven Nordic cohorts	No pollution and social factors were used for the empirical study.
	Association between secondhand smoke and cancers in adults in the US population	No pollution and social factors were used for the empirical study.
	Road and railway noise and risk for breast cancer: A nationwide study covering Denmark	No pollution and social factors were used for the empirical study.
	Outdoor light at night and breast cancer incidence in the Danish Nurse Cohort	No pollution and social factors were used for the empirical study.
	Identifying Lung Cancer Risk Factors in the Elderly Using Deep Neural Networks: Quantitative Analysis of Web-Based Survey Data	No pollution and social factors were used for the empirical study.
	Associations between calcium and magnesium intake and the risk of incident gastric cancer: A prospective cohort analysis of the National Institutes of Health-American Association of Retired Persons (NIH-AARP) Diet and Health Study	No pollution and social factors were used for the empirical study.
	Potential cancer risks associated with the consumption of raw, salted, and canned sardine contaminated by mercury, arsenic, lead, and cadmium in Egypt	Not an empirical study with cancer outcome reported.
	Carcinogenic effect of potential toxic metals in school children through contaminated drinking water around the industrial area of Pakistan	Not an empirical study with cancer outcome reported.
	PM2.5 and PM10-related carcinogenic and non-carcinogenic risk assessment in Iran	Not an empirical study with cancer outcome reported.
	Association of air pollution with ischemic heart disease, stroke, diabetes, COPD, lung cancer, and all-cause mortality: Effect modification by pro-inflammatory diet	Independent variables out of scope
	Comprehensive Analysis of Predictors and Outcomes in Breast Cancer Screening in Romania: Insights from Demographic, Clinical, and Lifestyle Factors	Independent variables out of scope
	Predictive Mortality and Gastric Cancer Risk Using Clinical and Socio-Economic Data: A Nationwide Multicenter Cohort Study	Not relevant
	Racial and urban-rural disparities in lung cancer care: Insight from a Latent Class Growth Analysis	Not an empirical study with cancer outcome reported
	Race and Ethnicity, Lifestyle, Diet, and Survival in Patients With Prostate Cancer	Independent variables out of scope
	Comprehensive Analysis of Predictors and Outcomes in Breast Cancer Screening in Romania: Insights from Demographic, Clinical, and Lifestyle Factors	Not an empirical study with cancer outcome reported
	Global, regional and national burden of lung cancer attributable to PM2.5 air pollution: Trends from 1990 to 2021 with projections to 2045	Not an empirical study with cancer outcome reported

	Environmental and Lifestyle Factors in the Onset of Pancreatic Cancer: A 10-Year Study from Kosovo as a Transitioning Country in Southeast Europe	Not an empirical study with cancer outcome reported
	Neighborhood socioeconomic disparities in cancer incidence following a hypothetical intervention to increase residential greenspace cover in the UK Biobank cohort	Independent variables out of scope
Full Paper screening	None rejected	

Table S3: Review summary of the publications studying the association of air pollution with cancer

Study	Country of origin	Study Objective	Study Design	Total Population (Female Population)	Concerned Contributing Factors	Health Outcome	Key Findings
Kayyal-Tarabeia et al., 2024[36]	Israel	Evaluating the associations between $PM_{2.5}$ and nitrogen oxides (NO_x) and cancer incidences.	Cohort Study	947,073 (465,190)	Air Pollution: $PM_{2.5}$, NO_x	All sites Cancer, Lung Cancer, Bladder Cancer, Breast Cancer, and Prostate Cancer	An increase in $PM_{2.5}$ and NO_x increased the cancer risks with statistical significance. The association of $PM_{2.5}$ with cancer was stronger than that with NO_x.
Lequy et al., 2023[134]	France	Analyzing the relationships between airborne metal $PM_{2.5}$ particles and cancer risks.	Cohort Study	All site Cancer:11,755 (2,631; Bladder Cancer:12,048; Lung Cancer: 12,059; Breast Cancer: 2,656 (2,656); Prostate Cancer: 9,321 (0)	Air Pollution: $PM_{2.5}$ (Arsenic, Cadmium, Chromium, Lead, Nickel, Vanadium)	All sites Cancer, Lung Cancer, Bladder Cancer, Breast Cancer, and Prostate Cancer	The increasing exposure to airborne metal $PM_{2.5}$ particles studied increased the cancer risks.
Roy et al., 2019[114]	Korea	Evaluating the carcinogenic risks from $PM_{2.5}$ and PM_{10} in the coal mining area of Jharia coalfield, India	Cross-Sectional Study		Air Pollution: $PM_{2.5}$, PM_{10}	Specific Cancer type not reported	The cancer risks from both PM_{10} and $PM_{2.5}$ were significant and exceeded the permissive USEPA's limits in the region.
Kang et al., 2015[155]	China	Evaluating the cancer risks from the polycyclic aromatic hydrocarbons (PAHs) from dust inhalation, ingestion and dermal contact in Hunan province, China	Cross-Sectional Study		Air Pollution: Polycyclic Aromatic Hydrocarbons	Specific Cancer type not reported	With a cancer incidence case per million people, a baseline acceptable level of risk, it was found that the cancer risks from PAHs via ingestion and dermal contact were higher than the acceptance level, with higher risks for children. However, the risks from inhalation were below the acceptable level.
Lev Bar-Or et al., 2023[152]	Israel	Evaluating adult-onset cancer risks in the Haifa Bay Area from adolescent exposure to industrial air pollution	Cohort Study	2,187,317 (892,747)	Air Pollution: Household air pollution	All Site Cancer, Melanoma, Leukemia, Thyroid, Central Nervous System (CNS) cancer, Breast Cancer, Head and Neck Cancer, Pulmonary Cancer, Reproductive (Female and Male) Cancer, Urinary Tract Cancer, Hodgkin's Lymphoma, Non-Hodgkin's Lymphoma	The increased exposure to industrial air pollutants led to an increased risk for 5 of the analyzed 13 cancer types (female breast cancer, CNS, leukemia, melanoma, and thyroid cancer) in the Haifa Bay area.
Sharma and Jain, 2020[156]	India	Evaluating the carcinogenic risks from the $PM_{2.5}$ bound Polycyclic	Cross-Sectional Study		Air Pollution: PAHs, $PM_{2.5}$	Specific cancer type not reported	Women cooking in an enclosed kitchen had higher cancer risks than in a semi-enclosed and open kitchen. The cancer risks were lower with the improved cookstoves than with the traditional ones.

		aromatic hydrocarbons exposure in women based on the kitchen characteristics and cookstove types					However, the risk was still above the USEPA standards.
Kalagbor Ihesinachi et al., 2019[154]	Nigeria	Evaluating the cancer risks from heavy metal exposure in soot in Port Harcourt, Nigeria	Cross-Sectional Study		Air Pollution: heavy metals in soot (Nickel, Iron, Lead, Cadmium, Chromium)	Specific cancer type not reported	The incremental lifetime cancer risks evaluated were within the USEPA acceptable limits.
Su et al., 2019[73]	Taiwan	Evaluating the association between air pollution and cancer incidence in Taiwan, from 2012 to 2016.	Ecological & Spatial Studies		Air Pollution: $PM_{2.5}$, PM_{10}, Sulphur Dioxide, Nitrogen Dioxide, Nitric Oxide, and Ozone	Specific cancer type not reported	Significant positive correlation was found for the association between all pollutants ($PM_{2.5}$, Sulphur Dioxide, Nitrogen Dioxide, Nitric Oxide, and Ozone) and cancer incident rates before Bonferroni correction. However, after the Bonferroni correction, only $PM_{2.5}$ was significantly correlated with cancer incidence.
Mungi et al., 2019[52]	USA	Evaluating the association between industrial benzene emissions and the 10-year incidence rates of cancers at the county level in Texas.	Ecological & Spatial Studies		Air Pollution: Benzene, $PM_{2.5}$	Lymphohematopoietic, lung, and lip cancers	A significant positive relation was found between $PM_{2.5}$ and cancer risks. However, no significant association was found between benzene exposure and cancer risk.
Cong, 2018[160]	China	Evaluating the association between the waste gas emissions from industries and cancer incidences in Shanghai, China	Ecological & Spatial Studies		Air Pollution: Waste gas from industries	Lip Cancer, Salivary gland Cancer, Oropharynx Cancer, Nasopharynx Cancer, Laryngopharynx Cancer, Esophagus Cancer, Stomach Cancer, Small intestine Cancer, Colorectal Cancer, Anus Cancer, Liver Cancer, Gallbladder Cancer, Pancreas Cancer, Paranasal sinus Cancer, Larynx Cancer, Thoracic organs Cancer, Bone Cancer, Melanoma of the skin Cancer, Connective and soft tissue Cancer, Breast Cancer, Cervix Cancer, Uterus Cancer, Ovary Cancer, Placenta Cancer, Prostate Cancer, Testis Cancer, Kidney Cancer, Bladder Cancer, Brain and	Significant positive association was found between waste gas and cancers on the salivary gland, small intestine, colorectal, anal, gall bladder, pancreas, thoracic organs, connective and soft tissue, prostate gland, kidney, bladder, thyroid, non-Hodgkin's lymphoma, lymphatic leukemia, myeloid leukemia, and other unspecified ones. However, a significant negative relation was found with esophageal cancer.

						CNS Cancer, Thyroid Cancer, Hodgkin's lymphoma Cancer, Non-Hodgkin's lymphoma Cancer, Lymphatic leukemia Cancer, Myeloid leukemia Cancer, and Other unspecified cancers	
Lequy et al., 2021[71]	France	Evaluating the associations between long-term exposure to black carbon (BC) and cancer risk in the French Gazel cohort	Cohort Study	19,348 (5,245)	Air Pollution: Black carbon, $PM_{2.5}$, NO_2	All Site Cancer and Lung Cancer	There was a significant association between black carbon and $PM_{2.5}$ exposure with increased all-site cancer risks.
Xiong et al., 2021[161]	Canada	Evaluating the cancer risks from the hazardous Volatile Organic Compounds (VOCs) in Calgary, Canada.	Cross-Sectional Study		Air Pollution: VOCs	Specific cancer type not reported	The cancer risks associated with VOC inhalation exceeded the acceptable risk level of Health Canada. The three most significant drivers of cancer risk were Carbon tetrachloride, benzene, and 1,3-butadiene.
Chen et al., 2022[174]	Netherlands	Evaluating the association of bladder cancer with long-term exposure to air pollution.	Cohort Study	302,493	Air Pollution: $PM_{2.5}$, Nitrogen dioxide, Black Carbon (BC), Ozone	Bladder Cancer	No significant association was found between $PM_{2.5}$ and Bladder cancer
Pedersen et al., 2018[175]	Denmark	Evaluate the association of bladder cancer with long-term exposure to ambient air pollution.	Cohort Study	303,431 (185,093)	Air Pollution: Nitrogen Oxides (NO_x), Nitrogen dioxide (NO_2), $PM_{2.5}$, $PM_{10,}$ Organic Carbon (OC)	Bladder Cancer	No significant association was found between Bladder cancer and any of the pollutants considered in the study.
Latifovic et al., 2015[151]	Canada	Evaluating the relationship between diesel exhaust exposure and bladder cancer.	Case-Control Study	658	Air Pollution: Diesel Exhaust	Bladder Cancer	Long-term exposure to higher concentrations of diesel exhaust for over 10 years was found to increase bladder cancer risk.
Locatelli et al., 2024[176]	Italy	To investigate the association of $PM_{2.5}$, PM_{10}, NO_2, and Formaldehyde with leukemia.	Case-Control Study	611 (349)	Air Pollution: $PM_{2.5}$, PM_{10}, NO_2, Formaldehyde	Leukemia	No significant association was found between the blood cancer and $PM_{2.5}$, PM_{10}, $NO_{2,}$ and Formaldehyde.
Taj et al., 2021[35]	Denmark	Investigation of the constituents of $PM_{2.5}$ responsible for Leukemia	Case-Control Study	66,596 (28,096)	Air Pollution: $PM_{2.5}$, BC, NH_4, NO_3, SO_4, Secondary inorganic aerosols (SIA), Secondary organic aerosols (SOA)	Leukemia	$PM_{2.5}$, NO_3, SIA, and NH_4 showed significant positive association with leukemia, whereas the association of BC, SO_4, and SOA was not significant.
Deziel et al., 2014[157]	USA	Investigating the association of PAHs exposure and risk of childhood acute lymphoblastic leukemia	Case-Control Study	629 (261)	Air Pollution: Polycyclic Aromatic Hydrocarbons (PAHs)	Leukemia	An increasing concentration of PAHs was associated with the risk of blood cancer. However, the significant association varied with the PAHs type. Indeno[1,2,3-cd] pyrene and Dibenzo[a,h]anthracene showed a significant association with the cancer risk.

Teras et al., 2019[163]	USA	Investigating the associations of benzene exposure and the risk of blood cancers.	Cohort Study	115,996 (63,442)	Air Pollution: Benzene	Blood Cancer	Significant associations between benzene and blood cancer were seen. But the significance depended on the type of blood cancer and sex. The association between benzene exposure and T-cell lymphoma was significant in both men and women.
Ward et al., 2014[177]	USA	Evaluating the association of Polybrominated diphenyl ethers (PBDEs) with acute lymphoblastic leukemia (ALL) incidence	Case-Control Study	381 (152)	Air Pollution: Polybrominated diphenyl ethers (PBDEs)	Acute Lymphoblastic Leukemia (ALL)	No significant association was discovered between total summed PBDEs and ALL. However, some non-common PBDEs like BDE-196, BDE-203, BDE-206, and BDE-207 showed a significant positive association with ALL.
Chen, 2024[131]	Canada	Investigating the association of residential Radon exposure and leukemia (chronic lymphocytic leukemia (CLL), non-Hodgkin's Lymphoma (NHL), Acute Lymphoblastic Leukemia (ALL), Myeloma, Others).	Ecological & Spatial Studies		Air Pollution: Radon	Leukemia	Radon exposure was significantly associated with Chronic Lymphocytic Leukemia (CLL) and non-Hodgkin lymphoma (NHL) in females. However, no significant association was found with other types.
Taj et al., 2024[34]	Denmark	Evaluating the relationship between long-term residential exposure to outdoor air pollution and risk of lymphoma and leukemia.	Cohort Study	276,978	Air Pollution: NO_2, $PM_{2.5}$, BC, O_3	Blood Cancer	Significant association was found for NO_2 and $PM_{2.5}$ with Leukemia and Lymphoma, respectively.
Hvidtfeldt et al., 2023[178]	Denmark	Investigating the association of Air pollution with Multiple myeloma risk	Cohort Study	234,803	Air Pollution: NO_2, $PM_{2.5}$, BC, O_3	Myeloma	No statistically significant association was discovered between the pollutants considered and multiple myeloma.
Khorrami et al., 2022[122]	Iran	Evaluating the association of ambient air pollution and leukemia (acute myeloid leukemia and lymphoid leukemia), in Tehran, Iran	Ecological & Spatial Studies		Air Pollution: PM_{10}, SO_2, NO_2, NO, NO_x, Benzene, Toluene, Ethylbenzene, p-xylene, o-xylene, m-xylene	Leukemia	Only NO_x showed a significant positive association with the blood cancer.
Lloyd et al., 2024[75]	Canada	Investigating the association of ultrafine particles (UFPs) with brain cancer in Toronto and Montreal	Cohort Study	1,544,000	Air Pollution: Ultrafine particles (UFPs), $PM_{2.5}$, Black Carbon (BC)	Malignant Brain Tumors	The long-term exposure to UFPs showed a consistent positive relation with brain cancer. However, other considered pollutants showed no such results.
Chang et al., 2022[76]	Taiwan	Evaluating the environmental risk factors (air	Cohort Study	161,213 (90,300)	Air Pollution: Carbon Monoxide (CO), NO_2, $PM_{2.5}$, SO_2, PM_{10}	Benign Brain Cancer	The long-term exposure to CO, NO_2, and $PM_{2.5}$ was significantly associated with benign brain cancer risk, whereas no significant association of the cancer was found with SO_2 and PM_{10}.

		pollutants) behind Benign brain cancer.					
Wu et al., 2020[77]	USA	Evaluating the associations between time-varying pollutants and the risk of malignant brain cancer and meningioma.	Cohort Study	103,308 (59,218)	Air Pollution: NO_2, NO_x, Benzene, $PM_{2.5}$, PM_{10}, CO, O_3	Malignant Brain Cancer	A significant association between the brain cancer risk and Benzene, PM_{10}, and O_3 was found for men. Latino men had the highest risk, whereas no significant relation was observed for women.
Harbo Poulsen et al., 2020[78]	Denmark	Evaluating the association of air pollutants with brain cancer	Case-Control Study	Brain Cancer:35,888; Benign Brain Cancer:9,182; Malignant Brain Tumors:26,706; Glioma: 20,158; Non-Glioma: 6,548	Air Pollution: $PM_{2.5}$, Organic Carbon, BC, SIA, SOA	Brain Cancer (Malignant, non-malignant, glioma and non-glioma)	A significant relationship between the carbon compounds and malignant brain cancer risk was observed. Furthermore, $PM_{2.5}$ and SIA were significantly associated with malignant non-glioma tumors.
Andersen et al., 2018[79]	Denmark	Evaluating the association of air pollutants with brain tumor risk	Cohort Study	Malignant Brain Tumors: 282,194; Benign Brain Tumors: 106,786; Brain Cancer: 388,980	Air Pollution: NO_2, NO_x, $PM_{2.5}$, PM_{10}, $PM_{2.5}$ absorbance, $PM_{2.5}$, Traffic intensity	Brain Cancer (Benign, Malignant, meninges)	No significant association was found between the pollutants considered and brain cancer.
Jørgensen et al., 2016[5]	Denmark	Investing in the relationship between air pollutants and brain tumor risk	Cohort Study	25,143	Air Pollution: NO_2, NO_x, $PM_{2.5}$, PM_{10},	Brain Cancer (Benign, Malignant, meninges)	No significant association was discovered between the pollutants and brain cancer.
Wu et al., 2021[144]	USA	Evaluating the association between airport-related ultra-fine particles (UFPs) and the risk of incident malignant brain cancer and meningioma	Cohort Study	75,936 (43,927)	Air Pollution: UFPs,	Brain Cancer	A significant association was found in African Americans between the increased risk of malignant brain cancer and UFPs exposure.
Weichenthal et al., 2020[143]	Canada	Evaluating the association of brain cancer hazard risk and exposure to ambient UFP	Cohort Study	1,938,100	Air Pollution: UFPs, NO_2, $PM_{2.5}$	Brain Cancer	A significant association was observed between ambient UFPs and brain cancer risk. However, no such association was observed with other considered pollutants.
Heck et al., 2024[166]	USA	Estimating the association of breast cancer risks to ambient air toxics exposure at residential addresses.	Cohort Study	48,665 (48,665)	Air Pollution: industry-related toxics, traffic-related toxics.	Breast Cancer	A significant association was discovered between the increased breast cancer risks and almost all the toxics studied, except for acrylonitrile and methyl isobutyl ketone, which showed no significant positive association.
Smotherman et al., 2023[58]	USA	Evaluating the association of air pollutant exposures and postmenopausal breast cancer risk.	Cohort Study	155,235 (155,235)	Air Pollution: NO_2, NO_x, $PM_{2.5}$, PM_{10}, $PM_{2.5\text{-}10}$, $PM_{2.5}$ absorbance	Breast Cancer	The increased exposure to PM_{10} was significantly associated with an increased breast cancer risk, whereas other considered pollutants showed no such association.
Rhee et al., 2023[167]	USA	Investigating the associations between proximity-based residential exposure to industrial emissions of dioxins and breast cancer risk	Cohort Study	35,908 (35,908)	Air Pollution: polychlorinated dibenzo-p-dioxins and dibenzofurans (PCDD/F)	Breast Cancer	Increased breast cancer risks were observed for increased exposure to emissions.
Li et al., 2021[59]	Taiwan	Evaluating the association of breast	Cohort Study	161,970 (161,970)	Air Pollution: NO_2, $PM_{2.5}$, CO, NO, Total	Breast Cancer	High exposure to all of the considered pollutants showed a significant association with breast cancer

		cancer risk and air pollution in Taiwan			Hydrocarbon (THC), Carbon tetrachloride (CH_4)		risks, with the strongest associations for THC and CH_4, except for $PM_{2.5}$.
White et al., 2021[60]	USA	Investigating the association of air pollution with breast cancer risk in black women.	Cohort Study	41,317 (41,317)	Air Pollution: NO_2, $PM_{2.5}$, O_3	Breast Cancer	No significant association was discovered between air pollution and increased cancer risk among black women. However, an increase in $PM_{2.5}$ exposure was significantly positively associated with breast cancer for women in the Midwest and inversely for those in the South.
Cheng et al., 2020[61]	USA	Evaluating the association of long-term Air Pollution exposure with breast cancer in the Southern California Multi-ethnic Cohort	Cohort Study	57,589 (57,589)	Air Pollution: NO_x, NO_2, $PM_{2.5}$, PM_{10}	Breast Cancer	Significant association was discovered between NO_x, NO_2, $PM_{2.5}$, PM_{10} and breast cancer risk and specifically for African American and Japanese American women.
Shmuel et al., 2017[147]	USA	Evaluating the relationship between childhood residential exposure to traffic-related air pollution with Breast cancer development	Cohort Study	42,934 (42,934)	Air Pollution	Breast Cancer	Women with postmenopausal breast cancer had a significantly higher risk if their nearest road had a median/barrier, and the ER-negative breast cancer risk was increased for roads with a median/barrier. Women living near a road with three or more lanes and a median/barrier also showed increased risk, with stronger trends in rural areas, though not statistically significant.
Andersen Zorana et al., 2017[62]	Denmark	Evaluating the association between the incidence of postmenopausal breast cancer and long-term exposure to ambient air pollution.	Cohort Study	68,806 (68,806)	Air Pollution: NO_2, NO_x, $PM_{2.5}$, PM_{10}, $PM_{2.5}$ absorbance, $PM_{2.5}$, Traffic intensity	Breast Cancer	No significant association was found between the considered pollutants and postmenopausal breast cancer.
Reding et al., 2015[63]	USA	Investigating the breast cancer risk associated with ambient air pollutants	Cohort Study	49,340 (49,340)	Air Pollution: NO_2, $PM_{2.5}$, PM_{10}	Breast Cancer	No significant association was discovered between the overall breast cancer risk and the considered pollutants.
Poulsen et al., 2023[32]	Denmark	Evaluating the association of breast cancer incidence with air pollutants	Case-Control Study	111,490 (111,490)	Air Pollution: NO_2, $PM_{2.5}$, Elemental Carbon	Breast Cancer	The considered pollutants were significantly associated with increased breast cancer risk.
Goldberg et al., 2019[116]	Canada	Investigating the association between ambient NO_2 and breast cancer risk.	Cohort Study	Post-menopausal Breast Cancer: 88,389 (88,389); Pre-menopausal Breast Cancer: 38,210 (38,210)	Air Pollution: NO_2	Breast Cancer	For premenopausal women, higher NO_2 exposure was significantly associated with increased breast cancer risk, but no such association was discovered for postmenopausal women.
White, et al., 2019[64]	USA	Investigating the relationship between PM components, air pollution, and breast cancer risk in the United States	Cohort Study	47,433 (47,433)	Air Pollution: NO_2, $PM_{2.5}$, PM_{10}	Breast Cancer	A significant association of overall breast cancer was discovered with NO_2, but not with $PM_{2.5}$ and PM_{10}.
Niehoff et al., 2019[172]	USA	Relation of mammary gland carcinogen classified air-toxics with breast cancer incidences.	Cohort Study	49,718 (49,718)	Air Pollution: 1,2-Dibromo-3-chloropropane, 1,3-Butadiene, 1,4-Dioxane, 2,4-Dinitrotoluene, 2,4-Toluene diisocyanate, 2-	Breast Cancer	A consistent association of methylene chloride was discovered with breast cancer risks. Association was discovered with other toxics like polycyclic organic matter, propylene dichloride, and styrene, too.

					Chloroacetophenone, Acrylamide, Acrylonitrile, Benzene, Benzidine, Carbon tetrachloride, Chloroprene, Ethylbenzene, Ethylene dibromide, Ethylene dichloride, Ethylene oxide, Ethylidene dichloride, Hydrazine, Methylene chloride, Nitrobenzene, o-Toluidine, Polycyclic organic matter, Propylene dichloride, Propylene oxide, Styrene, Toluene, Vinyl chloride, Vinylidene chloride, Xylenes		
Amadou et al., 2020[179]	France	Investigating the association of breast cancer risk (estrogen receptor negative/positive [ER−/ER+] and progesterone receptor negative/positive [PR−/PR+]) with long-term exposure to airborne cadmium pollution.	Cohort Study	9,058 (9,058)	Air Pollution: Cadmium	Breast Cancer	No significant association was discovered between the overall breast cancer risk and cadmium exposure. However, an inverse statistically significant association was discovered between ER-subtype and cadmium exposure.
White et al., 2019[173]	USA	Evaluating the association between metallic air pollutants and breast cancer risk.	Cohort Study	50,884 (50,884)	Air Pollution: Antimony, Arsenic, Cadmium, Chromium, Cobalt, Lead, Manganese, Mercury, Nickel, and Selenium	Breast Cancer	Mercury exposure was significantly associated with increased breast cancer risk. Whereas no such significant associations were found for other pollutants.
Andersen et al., 2017[113]	Denmark	Evaluating the relationship between long-term exposure to air particulate matter and breast cancer	Cohort Study	22,877 (22,877)	Air Pollution: NO_2, $PM_{2.5}$, PM_{10}	Breast Cancer	No significant association of breast cancer risk was observed for the considered pollutants.
Hart et al., 2016[65]	USA	Investigating the association between particulate matter (PM) and proximity to roads with breast cancer risk.	Cohort Study	115,921 (115,921)	Air Pollution: PM_{10}, $PM_{2.5–10}$, and $PM_{2.5}$	Breast Cancer	Postmenopausal breast cancer risk was inversely associated with $PM_{2.5}$ exposure. Furthermore, living near the major A1-A2 (A1: Major highways/freeways A2: Principal arterial roads) road was significantly associated with [ER−/ PR−] invasive breast cancer.
White et al., 2016[159]	USA	Investigating the association of breast cancer incidence and exposure to Polycyclic aromatic hydrocarbons (PAHs) from multiple sources	Case-Control Study	3,064 (3,064)	Air Pollution: PAHs	Breast Cancer	Breast cancer is associated with PAHs exposure from residential environmental tobacco smoke (ETS), synthetic fire log burning, and total indoor sources.

Garcia et al., 2015[165]	USA	Evaluating the relationship between modelled concentrations of air pollutants shown to be mammary gland carcinogens (MGCs) and breast cancer incidence.	Cohort Study	112,378 (112,378)	Air Pollution: Benzene, Methylene chloride, Carbon tetrachloride, 1,3-Butadiene, Styrene, Acrylonitrile, Vinyl chloride, Ethylidene dichloride, Ethylene oxide, Ethylene dichloride, 1,4-Dioxane, Propylene dichloride, Vinylidene chloride, Ethylene dibromide, Propylene oxide, 2,4-Toluene diisocyanate, Ethyl carbamate, Hydrazine, Chloroprene, Nitrobenzene, Acrylamide, 4,4'-Methylene bis(2-chloroaniline), o-Toluidine, Benzidine	Breast Cancer	The exposure to propylene oxide and vinyl chloride was found to be statistically significantly associated with breast cancer incidence. Furthermore, higher ambient levels of acrylamide, benzidine, carbon tetrachloride, ethylidene dichloride, and vinyl chloride were significantly associated with ER+/PR+ tumors and ER-/PR- tumors were associated with higher ambient levels of benzene.
Song et al., 2024[33]	China	Investigating the association between fine particulate matter and its components with breast cancer incidence in Beijing.	Cohort Study	85,504 (85,504)	Air Pollution: $PM_{2.5}$, NH_4, Organic Matter (OM), BC, SO_4, NO_3.	Breast Cancer	Increased exposure to all considered pollutants was significantly associated with breast cancer incidence.
White et al., 2024[66]	USA	Evaluating the association of outdoor fine particulate matter with breast cancer.	Cohort Study	196,905 (196,905)	Air Pollution: $PM_{2.5}$.	Breast Cancer	Overall, breast cancer incidence was significantly associated with $PM_{2.5}$. For subtype, ER+ was significantly associated with $PM_{2.5}$ exposure, whereas ER- was not.
Bai et al., 2020[67]	Canada	Evaluating the association of long-term exposure to air pollutants with lung cancer and breast cancer.	Cohort Study	Lung Cancer: 4,952,022 (2,564,340); Breast Cancer:2,564,340 (2,564,340)	Air Pollution: $PM_{2.5}$, NO_2, O_3	Lung Cancer, Breast Cancer	A non-significant positive association was discovered for $PM_{2.5}$, NO_2 and lung cancer, whereas significant associations were observed for ozone, showing reduced cancer risks. In addition, no association were found between breast cancer and air pollutants.
Hu et al., 2023[153]	China	Investigating the association of air pollution with breast cancer and cervical cancer.	Ecological & Spatial Studies		Air Pollution: Particulate Matter (PM), Soot	Breast Cancer, Cervical Cancer	An inverse non-linear relationship between soot emissions and both cervical and breast cancer was discovered. Furthermore, a significant association was discovered with the number of medical institutions and health staff.
J. M. Lee et al., 2022[72]	South Korea	Investigating the association of childhood cancer with $PM_{2.5}$	Cohort Study	1,261,855 (610,151)	Air Pollution: $PM_{2.5}$, PM_{10}, SO_2, NO_2, CO, O_3	Childhood cancer: Lymphoid, Leukemia, Myeloid Leukemia, Brain Cancer	$PM_{2.5}$ was found to be significantly associated with childhood cancers, lymphoid leukemia, hematopoietic cancers and CNS cancers, and ozone was found to be significantly associated with myeloid leukemia. However, no significant association was found for other considered pollutants.
Lavigne et al., 2020[140]	Canada	Investigating the relationship between childhood cancer development with prenatal and childhood exposure to UFPs.	Cohort Study	653,702	Air Pollution: UFPs (<0.1μm)	All: Childhood Cancer Acute Lymphoid Leukemia, Astrocytoma, Neuroblastoma	A significant association of first-trimester exposure to UFPs with childhood cancer was discovered.

Lavigne et al., 2017[80]	Canada	Investigating the association of pediatric cancers with maternal exposure to ambient air pollutants	Cohort Study	2,350,898	Air Pollution: NO_2, $PM_{2.5}$,	Childhood cancer, Acute Lymphoid Leukemia, Wilms Tumor, Astrocytoma	A significant association was found for $PM_{2.5}$ exposure during the first trimester and astrocytoma, NO_2 exposure and all types of cancer, and astrocytoma specifically.
Hüls et al., 2023[149]	USA	Assessing the association of childhood cancer with air pollution and socioeconomic factors.	Ecological & Spatial Studies		Air Pollution, Socioeconomic factors	Childhood cancer: International Classification of Childhood Cancer (ICCC). Class I Leukemias, Myeloproliferative and Myelodysplastic Diseases. Class II Lymphomas and Reticuloendothelial Neoplasms. Class III CNS and Miscellaneous Intracranial and Intraspinal Neoplasms.	A significant association was found for Lymphoma risk with high pesticide exposure and low socioeconomic status in rural areas. Furthermore, counties with lower alcohol consumption had significantly lower lymphoma risk. However, no significant associations were found for Leukemia and CNS.
Spycher et al., 2015[145]	Switzerland	Assessing the association of pediatric cancer risks with residential exposure to highways	Cohort Study	2,096,402	Air Pollution: Traffic proximity	Childhood cancer: leukemia, Acute Lymphoid leukemia, lymphoma, CNS tumors, and all other cancers	A statistically significant association was discovered for children under 5 living <100m from a highway with leukemia. Furthermore, lymphoma is significantly associated with higher traffic volume (an additional 10,000 vehicles/day).
Zhang et al., 2020[162]	China	Evaluating the association of indoor air pollution and childhood acute leukemia (AL) in Shanghai.	Case-Control Study	245 (78)	Air Pollution: VOCs	Childhood cancer, Acute Leukemia, Acute Lymphoblastic Leukemia, Acute Myeloid Leukemia	A significant association of acute leukemia with exposure to styrene and butyl alcohol was found.
Hvidtfeldt et al., 2020[95]	Denmark	Investigating the association of exposure to $PM_{2.5}$ components with childhood non-Hodgkin lymphoma (NHL)	Case-Control Study	779 (241)	Air Pollution: $PM_{2.5}$, SOA, SIA, BC, OC, sea salt	Blood Cancer	$PM_{2.5}$ and BC exposure were significantly associated with NHL in children. However, no significant associations were discovered for other pollutants considered in the study.
Danysh et al., 2016[146]	USA	Investigating the relationship between childhood central nervous system (CNS) tumors and maternal residential proximity to major roadways	Case-Control Study	1,890 (925)	Air Pollution, Roadway Proximity	CNS cancer Astrocytoma (non-Juvenile pilocytic astrocytoma, non-JPA) JPA, Ependymoma, Medulloblastoma, Primitive neuroectodermal tumor (PNET)	All CNS tumors' risk in children increased significantly with an increase in the nearness of the maternal residency to a major roadway. In addition, high roadway density was also significantly related to all CNS cancer incidence in children.
Hvidtfeldt et al., 2023[82]	Denmark	Evaluating the association between residential exposure to air pollutants and malignant	Cohort Study		Air Pollution: NO_2, $PM_{2.5}$, BC, O_3	CNS cancer	No statistically significant associations were discovered between the assessed air pollutants and malignant intracranial CNS tumors.

		intracranial CNS tumors.					
Aslak Harbo et al., 2020[81]	Denmark	Evaluating the association of residential outdoor exposure and CNS tumors.	Case-Control Study	58,425	Air Pollution: NO_x, $PM_{2.5}$, BC, O_3	CNS Cancer (Non-Malignant Non-Glioma Brain Tumors, Malignant Non-Glioma Brain Tumors, Cranial Nerve Tumors, Meningioma, Glioma)	Significant positive associations between malignant intracranial CNS tumors and BC, malignant non-glioma brain tumors, $PM_{2.5}$, meningioma, and NO_2 were discovered. However, O_3 is significantly inversely related to Meningioma.
Jiang et al., 2024[148]	China	Evaluating the association of air pollutants using the air pollutants exposure score (APES) with Colorectal Cancer.	Cohort Study	428632	Air Pollution: NO_x, $PM_{2.5}$	Colorectal Cancer	A higher APES score was significantly associated with increased Colorectal Cancer risk.
Chu et al., 2021[89]	China	Evaluating the relation between $PM_{2.5}$ exposure, genetic variants, and colorectal cancer risk.	Cohort Study	139,534 (69,625)	Air Pollution: $PM_{2.5}$	Colorectal Cancer	$PM_{2.5}$ exposure was significantly associated with an increased risk of colorectal cancer.
Sun et al., 2023[91]	China	Evaluating the association of long-term exposure to $PM_{2.5}$ and esophageal cancer incidence.	Cohort Study	510,125 (300,900)	Air Pollution: $PM_{2.5}$	Esophageal Cancer	$PM_{2.5}$ exposure (per 10 $\mu g/m^3$ increase) was significantly associated with increased Esophageal cancer risk.
Nagel et al., 2024[92]	Germany	Evaluating the association of Air pollution exposure with upper aerodigestive tract (UADT) cancer and gastric cancer	Cohort Study	Gastric Cancer: 333,525; UADT Cancer: 343,625	Air Pollution: NO_2, $PM_{2.5}$, BC, Ozone in the warm season (O_{3w})	UADT Cancer and Gastric Cancer	No significant association was found for air pollutants considered and Gastric cancer. However, UADT cancer showed an increased risk with NO_2, BC, and $PM_{2.5}$. Furthermore, a significant inverse association was discovered for O_{3w} exposure.
Weinmayr et al., 2018[102]	Germany	Investigating the association between long-term exposure to elemental components of $PM_{2.5}$ and PM_{10} and gastric and UADT cancer incidence.	Cohort Study		Air Pollution: $PM_{2.5}$, PM_{10}	UADT Cancer and Gastric Cancer	Sulphur in $PM_{2.5}$ was found to be significantly associated with gastric cancer, and no other significant associations were reported.
Chen et al., 2023[103]	China	Assessing the association of air pollutants and gastrointestinal cancer incidence	Cohort Study	4,708 (2,265)	Air Pollution: $PM_{2.5}$, PM_{10}, NO_x, NO_2	Gastrointestinal cancer	No significant association was discovered between exposure to the considered air pollutants and gastrointestinal cancer.
Hvidtfeldt et al., 2022[104]	Denmark	Evaluating the association of Air pollutants with kidney parenchyma cancer	Cohort Study	302,493	Air Pollution: NO_2, $PM_{2.5}$, BC, Ozone in the warm season (O_{3w})	Kidney Cancer	No significant association of the considered air pollutants with Kidney parenchyma cancer was observed.
Raaschou-Nielsen et al., 2017[105]	Denmark	Evaluating the associations between outdoor air pollution	Cohort Study	289,002	Air Pollution: NO_2, NO_x, $PM_{2.5}$, PM_{10}, $PM_{2.5}$ absorbance, $PM_{2.5}$	Kidney cancer	No significant associations were discovered for the considered air pollutants and kidney parenchyma cancer.

		at the residence and the incidence of kidney parenchyma cancer in the general population.					
Wang et al., 2023[119]	China	Evaluating the association of long-term exposure to ambient air pollutants and laryngeal cancer risks.	Cohort Study	418,914	Air Pollution: NO_2, NO_x, $PM_{2.5}$, PM_{10}	Laryngeal Cancer	Higher exposure to NO_2, NO, and $PM_{2.5}$ were significantly related to increased laryngeal cancer risks.
Lu et al., 2024[84]	Taiwan	Investigating the association of long-term exposure to metal constituents in $PM_{2.5}$ with the risk of liver cancer using a Taiwanese cohort study	Cohort Study	13,511	Air Pollution: $PM_{2.5}$	Liver Cancer (Hepatocellular Carcinoma)	Exposure to $PM_{2.5}$ copper was significantly associated with higher liver cancer risk.
VoPham et al., 2018[85]	USA	Evaluating the relation of $PM_{2.5}$ exposure and liver cancer risk in the US.	Cohort Study	56,245	Air Pollution: $PM_{2.5}$	Liver cancer (Hepatocellular Carcinoma)	Increased $PM_{2.5}$ exposure was significantly associated with increased hepatocellular cancer risk.
Pedersen et al., 2017[86]	Denmark	Evaluating the association between residential exposure to air pollution and liver cancer incidence.	Cohort Study	174,770	Air Pollution: NO_2, NO_x, $PM_{2.5}$, PM_{10}, $PM_{2.5\ absorbance}$	Liver cancer	No statistical association was observed between the considered air pollutants and liver cancer.
Pan et al., 2016[87]	Taiwan	Evaluating the association of long-term exposure to $PM_{2.5}$ and risk of hepatocellular carcinoma.	Cohort Study	22,062	Air Pollution: $PM_{2.5}$	Liver Cancer (Hepatocellular Carcinoma)	Long-term exposure to $PM_{2.5}$ was significantly associated with hepatocellular carcinoma risk.
Ma et al., 2024[88]	USA	Investigating the association of $PM_{2.5}$ exposure with liver cancer incidence.	Cohort Study	49,929	Air Pollution: $PM_{2.5}$	Liver Cancer (Hepatocellular Carcinoma)	No significant association was found between $PM_{.5}$ exposure and liver cancer
Deen et al., 2022[171]	Denmark	Evaluating the association of airborne polychlorinated biphenyls (PCBs) exposure with cancer risk.	Cohort Study	38,613	Air Pollution: PCBs	All cancers (minus nonmelanoma skin), Esophageal Cancer, Colon Cancer, Rectal Cancer, Liver Cancer, Pancreatic Cancer, Lung Cancer, Melanoma, Nonmelanoma skin cancers, Breast Cancer, Cervix uteri, Prostate Cancer, Urinary bladder, Meningiomas, Brain,	Significant associations were observed for both PCB exposure greater than 2.9 years and concentration greater than 3000 ng/m^3 with liver cancer, meningiomas, and pancreatic cancer. Moreover, both short-term exposure (less than or equal to 1 year) and moderate concentration (300- 949ng/m^3) of PCBs were significantly associated with Testicular cancer.

						Ill-defined and unspecified cancer	
Bookstein et al., 2024[141]	USA	Evaluating the association of long-term exposure to airport-related ultrafine particles (UFPs) with lung cancer	Cohort Study	71,387 (41,413)	Air Pollution: UFPs	Lung Cancer (Adenocarcinoma, Small Cell carcinomas, Large Cell Carcinoma, squamous cell carcinoma)	Lung cancer was not significantly associated with long-term UFP exposure.
Jones et al., 2023[142]	USA	Evaluating the relationship between UFP and lung cancer overall and by histologic subtype.	Cohort Study	1,770	Air Pollution: UFPs	Lung Cancer Adenocarcinoma, Small Cell carcinomas, squamous cell carcinoma)	No significant association was discovered between overall lung cancer and UFP exposure; however, a significant association was observed for adenocarcinoma and UFP in males.
Li et al., 2023[45]	China	Exploring the air pollution risk factors behind lung cancer in North China.	Case-Control Study	14,604 (6,356)	Air Pollution: $PM_{2.5}$	Lung Cancer	$PM_{2.5}$ was found to be significantly associated with lung cancer incidence.
Liang et al., 2023[37]	China	Assessing the relation of air pollution with lung cancer.	Cohort Study	36,723 (175,247)	Air Pollution: $PM_{2.5}$, PM_{10}, NO_2, NO_x	Lung Cancer	Long-term exposure to $PM_{2.5}$, PM_{10}, NO_2, and NO_x was significantly associated with lung cancer.
N.-W. Lee et al., 2022[135]	Taiwan	Evaluating the association of airborne heavy metals with lung cancer.	Cohort Study	3,390	Air Pollution: Heavy metals (Vanadium, Chromium, Manganese, Nickel, Copper, Arsenic, Strontium, Cadmium, Mercury, Thallium, Lead)	Lung Cancer (Adenocarcinoma, Small Cell carcinomas, squamous cell carcinoma)	A higher exposure to cadmium was significantly associated with lung cancer.
Huang et al., 2021[38]	Taiwan	Evaluating the association of smoking, sex, lifestyle, diet and $PM_{2.5}$ with lung cancer risk.	Cohort Study	174,431 (87,722)	Air Pollution: $PM_{2.5}$	Lung Cancer	Age older than 50, lower education level, ever smokers, and female never smokers had significantly higher risks of developing lung cancer. Furthermore, for never smokers, $PM_{2.5}$ exposure significantly raised lung cancer risk. In addition, consuming more than two servings of vegetables and fruits reduced the lung cancer risk significantly.
Kamis et al., 2021[138]	USA	Preparing and comparing multiple models to predict lung cancer in the USA.	Ecological & Spatial Studies		Air Pollution: Adult smoking, state, environmental quality index, and ambient emissions: Cyanide (CN) compounds, C_O, CS_2, Diesel Exhaust, NO_2, O_3, $PM_{2.5}$, PM_{10} and SO_2	Lung Cancer	Multiple models were formulated and compared to find out which is the best to predict lung cancer. Based on the best-performing model, $PM_{2.5}$, NO_2, and SO_2 were the most significant emissions contributing towards lung cancer. Adult smoking was found to be the most dominant one, and next were the states. The Sociodemographic Environmental Quality Index (EQI) was significantly negatively related to lung cancer.
Yang et al., 2021[106]	South Korea	Assessing the association of long-term exposure to particulate matter and nitrogen dioxide with lung cancer.	Cohort Study	83,478	Air Pollution: $PM_{2.5}$, PM_{10}, NO_2	Lung Cancer	No significant association was discovered between the considered pollutants and lung cancer.
Hvidtfeldt et al., 2021[39]	Denmark	Assessing the associations between long-term low-level air pollution exposure and lung cancer incidence.	Cohort Study	307,550	Air Pollution: $PM_{2.5}$, NO_2, O_{3w}, BC	Lung Cancer (Adenocarcinoma, Squamous Cell Carcinoma)	Increase in $PM_{2.5}$ exposure was significantly associated with lung cancer risk, whereas no such association was discovered for other pollutants in the final model.

Moon et al., 2020[110]	South Korea	Studying the association of ambient air pollution exposure and lung cancer incidence by histological subtype.	Cohort Study	6,567,909 (2,253,429)	Air Pollution: PM_{10}, NO_2	Lung Cancer (Non-small cell carcinoma, Adenocarcinoma, squamous cell carcinoma, large cell carcinoma, Small Cell carcinoma)	PM_{10} exposure significantly increased the risk of adenocarcinoma in male current smokers. However, it was associated with a decreased risk of squamous cell carcinoma in female current smokers. Furthermore, NO_2 exposure was significantly associated with decreased risk of lung cancer (non-small cell carcinoma and squamous cell carcinoma) in male non-smokers and with the Risk of small cell carcinoma in female non-smokers.
Gharibvand et al., 2017[40]	USA	Examining the association of $PM_{2.5}$ exposure and lung cancer incidence.	Cohort Study	80,285	Air Pollution: $PM_{2.5}$, O_3	Lung Cancer (Adenocarcinoma, Squamous cell carcinoma, large cell carcinoma, Small Cell carcinoma)	$PM_{2.5}$ exposure was significantly associated with lung cancer incidence.
Tomczak et al., 2016[41]	Canada	Assessing the association of long-term $PM_{2.5}$ exposure and Lung Cancer in Canada	Cohort Study	89,234 (89,234)	Air Pollution: $PM_{2.5}$	Lung Cancer (Adenocarcinoma, Squamous cell carcinoma, large cell carcinoma, Small Cell carcinoma)	Long-term $PM_{2.5}$ exposure was associated with lung cancer. Furthermore, the strongest association was discovered between small cell carcinoma and adenocarcinoma.
Raaschou-Nielsen et al., 2016[107]	Denmark	Examining the association of particulate matter (PM) exposure and lung cancer.	Cohort Study	245,782	Air Pollution: PM	Lung Cancer	No statistically significant associations were discovered for the PM exposure and Lung cancer incidences; however, when the model was restricted to the population who did not change their address during the study period. Significant associations were found for Sulphur, Copper, Nickel, Potassium, and Zinc particulate matter components.
Hart Jaime et al., 2015[42]	USA	Assessing the relationship between pollution and incident lung cancer in the Netherlands	Cohort Study	7,881	Air Pollution: NO_2, $PM_{2.5}$, Black Smoke	Lung Cancer (Adenocarcinoma, Squamous cell carcinoma, large cell carcinoma, Small Cell carcinoma)	Black smoke and NO_2 were found to be significantly associated with all lung cancer risk.
Puett Robin et al., 2014[108]	USA	Evaluating the association of long-term residential exposures to ambient particulate matter and residential roadway proximity with lung cancer.	Cohort Study	103,650	Air Pollution: $PM_{2.5}$, $PM_{2.5-10}$, PM_{10}	Lung Cancer	No significant association was observed for the considered variables, however, PM_{10} and $PM_{2.5}$ exposure were significant when the population was restricted to those who had not smoked for at least 10 years.
Xing et al., 2019[53]	China	Evaluating the non-linear spatial association between air pollutants and lung cancer incidence in males and females in North and South China	Ecological & Spatial Studies		Air Pollution: $PM_{2.5}$, SO_2, PM_{10}, CO, O_3 and NO_2	Lung Cancer	In North China, SO_2 and smoking were significant factors of lung cancer in males and females, respectively. Furthermore, in South China, $PM_{2.5}$, SO_2, NO_2, O_3, CO, and smoking were significant in males, whereas, $PM_{2.5}$, PM_{10}, and CO were significant factors in females.
Gowda et al., 2019[57]	USA	Evaluating the association of ambient air pollution and lung cancer in	Cohort Study	65,419 (65,419)	Air Pollution: $PM_{2.5}$, NO_2	Lung Cancer (Adenocarcinoma)	No significant association was discovered for the association of lung cancer risk and ambient air pollutants in women who have never smoked.

		women who have never smoked.					
Hughes et al., 2019[31]	USA	Assessing the relation of air quality indices with lung cancer in all Texas counties.	Ecological & Spatial Studies		Air Pollution: $PM_{2.5}$, Radon levels	Lung Cancer	Only increased $PM_{2.5}$ exposure was significantly related to lung cancer.
Lorenzo-González et al., 2019[125]	Spain	Examining the association of residential radon exposure and lung cancer in the northwest of Spain.	Case-Control Study	1,415 (887)	Air Pollution: Radon	Lung Cancer (Adenocarcinoma)	Increased radon exposure was significantly associated with lung cancer and subtype adenocarcinoma.
Dempsey et al., 2018[127]	Ireland	Investigating the relationship between radon exposure and lung cancer	Cross-Sectional Study	5,590	Air Pollution: Radon	Lung Cancer	No significant association was discovered for Radon exposure was related to lung cancer.
Lamichhane et al., 2017[109]	South Korea	Examining the association between lung cancer and long-term exposure to ambient air pollution in South Korea.	Case-Control Study	1,816 (684)	Air Pollution: NO_2, PM_{10}	Lung Cancer (Adenocarcinoma, Squamous cell carcinoma, large cell carcinoma, Small Cell carcinoma)	Increased PM_{10}, NO_2 exposure was found to be significantly associated with lung cancer risks. Furthermore, exposure to NO_2 was significantly related to an increase in small cell carcinoma.
Gharibvand et al., 2017[123]	USA	Examining the association between ambient $PM_{2.5}$ and incident lung adenocarcinoma risk in Non-smokers	Cohort Study	80,044 (27,868)	Air Pollution: $PM_{2.5}$, O_3	Lung Cancer (Adenocarcinoma)	No significant association was discovered; however, when the skin cancer population was excluded, the lung cancer risk was found to be significantly associated with the $PM_{2.5}$ exposure.
Han et al., 2016[158]	China	Evaluating the cancer risk associated with exposure to particulate carcinogenic PAHs for the elderly population in Tianjin, China	Risk Assessment & Modelling	80 (34)	Air Pollution: PAHs	Lung Cancer	The inhalation lifetime cancer risk (ICLR) associated with PAHs exceeded the USEPA acceptable levels for both men and women.
Raspanti et al., 2016[168]	USA	Examining the relationship between lung cancer risk and household air pollution (HAP) resulting from the combustion of biomass in the never-smoking Nepalese population	Case-Control Study	1,212 (494)	Air Pollution: HAP	Lung Cancer	HAP exposure was significantly associated with lung cancer. Furthermore, lung cancer was statistically associated with tobacco use, old age, and being female. However, the risk decreased significantly with higher socioeconomic status.
Guo et al., 2016[43]	Australia	Evaluating the relationship between lung cancer incidence and $PM_{2.5}$ and O_3 in China	Ecological & Spatial Studies	368,762 (121,229)	Air Pollution: $PM_{2.5}$, O_3	Lung Cancer	A significant positive association between overall lung cancer risk with $PM_{2.5}$ and O_3 was found. The cancer risk was significantly associated with both $PM_{2.5}$ and O_3 in males, females, Urban, age 30-65, age 65-75, and age >75.

Lee et al., 2015[126]	South Korea	Assessing the association of radon exposure with lung cancer risk in South Korea.	Ecological & Spatial Studies		Air Pollution: Radon	Lung Cancer	Radon exposure was attributable to 13.5%-19.5% of lung cancer deaths in males and 20.4%-28.2% in females.
Chen, et al., 2024[44]	Netherlands	Evaluating the relation between long-term air pollution exposure and lung cancer incidence.	Cohort Study	7,657,323	Air Pollution: $PM_{2.5}$, NO_2, BC, O_{3w}	Lung Cancer	A positive significant association was observed for lung cancer incidence and exposure to $PM_{2.5}$, NO_2, and BC. However, O_{3W} exposure showed a significant inverse relation.
Lin et al., 2024[54]	Taiwan	Evaluating the association of $PM_{2.5}$ exposure and adenocarcinoma incidences in Taiwan.	Case-Control Study		Air Pollution: $PM_{2.5}$	Lung Cancer (Adenocarcinoma)	Adenocarcinoma incidence increased significantly with the increase in $PM_{2.5}$ exposure.
Liu et al., 2023[46]	USA	Evaluating the association of $PM_{2.5}$, NO_2, O_{3w}, and particle radioactivity (PR) exposures on lung cancer incidence in older Americans (≥ 65 years)	Cohort Study	12,429,951	Air Pollution: $PM_{2.5}$, NO_2, O_{3w}, and particle radioactivity (PR)	Lung Cancer	Increased lung cancer risk was significantly associated with increased $PM_{2.5}$ and NO_2 exposures, while O_{3W} was significantly and inversely associated with the lung cancer risk, and PR was marginally significantly associated.
Cierpiał-Wolan et al., 2023[47]	Poland	Evaluating the association of long-term exposure to air pollutants and lung adenocarcinoma.	Cohort Study		Air Pollution: NO_2, $PM_{2.5}$, PM_{10}, SO_2, CO	Lung Cancer (Adenocarcinoma)	For the older population (>70 years), $PM_{2.5}$, PM_{10}, SO_2, and CO were significantly positively associated with lung cancer risk. Moreover, all of the considered pollutants were significantly positively associated with lung cancer risk for the population below the age of 70 years.
Gawełko et al., 2022[51]	Poland	Evaluating the association of long-term exposure to air pollutants and lung cancer incidence(squamous) in south-eastern Poland.	Cohort Study	4,237	Air Pollution: NO_2, $PM_{2.5}$, PM_{10}, SO_2,	Lung Cancer (Squamous Cell Cancer)	A positive association between the cancer risk and exposure to all pollutants was seen in women. Whereas for men significant inverse relation was seen for the population above 75 years or NO_2 and $PM_{2.5}$ exposure. For the general population below the age of 75, a significant positive association was seen with NO_2 and PM_{10} exposure.
Coleman Nathan et al., 2020[68]	USA	Evaluating the association between the incidence of cancer and exposure to $PM_{2.5}$	Ecological & Spatial Studies		Air Pollution: $PM_{2.5}$	All Cancer, Oral Cancer, Esophageal Cancer, Stomach Cancer, Small Intestine Cancer, Colon Cancer, Rectal Cancer, Liver Cancer, Pancreatic Cancer, Nose Cancer, Laryngeal Cancer, Lung Cancer, Bone Cancer, Skin Cancer, Soft Tissue Cancer, Breast Cancer, Cervical Cancer, Uterine Cancer,	Significant positive association was discovered for all cancers as a whole, lung cancer, Rectal Cancer, Liver Cancer, Nasal Cancer, Skin Cancer, Breast Cancer, Renal Cancer, and Endocrine Cancer with $PM_{2.5}$ exposure.

						Ovarian Cancer, Prostate Cancer, Other Male-Specific Cancers, Kidney Cancer, Bladder Cancer, Brain Cancer, Endocrine Cancer, Ill-Defined Cancers	
Lorenzo-Gonzalez et al., 2020[124]	Spain	Investigating the relationship between radon exposure and lung cancer risk.	Case-Control Study	3,702 (1,216)	Air Pollution: Radon	Lung Cancer	Increased radon exposure was significantly associated with lung cancer.
Li et al., 2020[48]	China	Evaluating the response pattern of lung cancer associated with high $PM_{2.5}$ exposure	Cohort Study	118,551	Air Pollution: $PM_{2.5}$	Lung Cancer	Increased $PM_{2.5}$ exposure was significantly and positively related to increased lung cancer risks.
Guo et al., 2020[6]	China	Investigating the association of $PM_{2.5}$ exposure with lung cancer risk for males and females in China	Ecological & Spatial Studies		Air Pollution: $PM_{2.5}$	Lung Cancer	Significant association of $PM_{2.5}$ exposure with lung cancer was discovered for both males and females.
Nikkilä et al., 2020[130]	Finland	Predicting indoor radon concentration and evaluating its association with childhood leukemia.	Case-Control Study	244,059	Air Pollution: Radon	Leukemia	No significant association of Radon exposure was discovered with childhood leukemia incidence.
Lim et al., 2023[49]	Australia	Investigating the associations of air pollutants with lung cancer and bladder cancer in a low-pollution city.	Cohort Study	Lung Cancer: 11,617; Bladder Cancer: 11,627	Air Pollution: $PM_{2.5}$, NO_2, BC	Lung Cancer and Bladder Cancer	$PM_{2.5}$ and BC were found to be significantly associated with lung cancer in a single pollutant model. In addition, BC was associated significantly with lung cancer in the two-pollutant model. However, no significant association was discovered for bladder cancer.
Wang et al., 2019[55]	China	Assessing the urban-rural and sex differences in the relation of $PM_{2.5}$ with the ten most common cancers in China in the southeastern side of the Hu line.	Ecological & Spatial Studies		Air Pollution: $PM_{2.5}$	Lung cancer, Ovarian Cancer, Prostate Cancer, Blood Cancer	$PM_{2.5}$ exposure was significantly associated with lung cancer, prostate cancer, leukemia, and ovarian cancer. In males, Prostate cancer, Leukemia, Brain cancer, Pancreatic cancer, Bladder cancer, and Colorectal cancer were significant, and for females, Lung cancer, Breast cancer, Colorectal cancer, Uterine cancer, Ovarian cancer, and Cervical cancer were significant. Furthermore, ovarian and prostate cancer risks are higher in urban areas, and lung cancer and leukemia risks are significantly higher in rural areas.
So et al., 2021[83]	Denmark	Evaluating the association between air pollution and liver cancer incidence.	Cohort Study	330,064	Air Pollution: NO_2, $PM_{2.5}$, O_{3w}, BC	Liver Cancer	NO_2, $PM_{2.5}$ components: Sulphur and Vanadium were significantly and positively associated with liver cancer incidence. However, Ozone was inversely significantly associated with liver cancer.
Ha et al., 2017[128]	South Korea	Investigating the association of Indoor radon exposure with Lung cancer, non-Hodgkin lymphoma, and Leukemia in Korea	Ecological & Spatial Studies		Air Pollution: Radon	Lung Cancer, Blood Cancer (Hodgkin Lymphoma, Non-Hodgkin Lymphoma)	Significant associations were discovered for lung cancer in males and non-Hodgkin lymphoma in females for radon exposure.

Diver et al., 2024[120]	Spain	Assessing the association of hematologic cancer with air pollution	Cohort Study	108,002 (56,936)	Air Pollution: $PM_{2.5}$, PM_{10}, $PM_{2.5-10}$, NO_2, O_3, SO2, and CO	Blood cancer (Hodgkin Lymphoma, Non-Hodgkin lymphoma, Myeloid Leukemia)	Exposure to NO_2 is significantly positively associated with mantle cell lymphoma and marginal zone lymphoma. A similar association was observed between CO and Marginal Zone lymphoma, mantle cell lymphoma, and $PM_{10-2.5}$. However, a significant inverse association was observed for PM_{10} and NO_2 with chronic lymphocytic leukemia/small lymphocyte lymphoma (CLL/SLL).
Chen, et al., 2024[169]	USA	Examining the associations of residential exposure to HAPs at the census tract level with multiple myeloma and non–Hodgkin lymphoma (NHL) incidences	Cohort Study	204,788 (204,788)	Air Pollution: HAP	Blood Cancer (Non-Hodgkin Lymphoma, Multiple Myeloma)	No significant associations were discovered.
Ferrante et al., 2016[170]	Italy	Evaluating the association between asbestos cumulative exposure and pleural malignant mesothelioma (PMM).	Case-Control Study	548 (201)	Air Pollution: Asbestos	Mesothelioma cancer	Increasing asbestos exposure increases cancer risks significantly. A similar trend was seen for subjects who were never occupationally exposed or domestically exposed.
Kentros et al., 2024[100]	USA	Examining the relation to investigate the association between $PM_{2.5}$ and ovarian cancer incidence in the USA.	Ecological & Spatial Studies	98,751	Air Pollution: $PM_{2.5}$	Ovarian Cancer	Increased exposure to $PM_{2.5}$ was significantly associated with ovarian cancer.
Bhavsar et al., 2024[98]	USA	Assessing the relationship between $PM_{2.5}$ and Pancreatic cancer	Case-Control Study	5,230 (3,231)	Air Pollution: $PM_{2.5}$	Pancreatic Cancer	Increased exposure to $PM_{2.5}$ was statistically associated with increased Pancreatic cancer risk.
Bogumil et al., 2021[99]	USA	Evaluating the association of air pollutants with pancreatic cancer	Cohort Study	100,527	Air Pollution: NO_2, $PM_{2.5}$, PM_{10}, NO_x	Pancreatic Cancer	A significant association was found between $PM_{2.5}$ exposure and increased pancreatic cancer risk in the general public. Furthermore, this association was significant for Latinos, women, ever smokers, those who moved, and individuals with a BMI of 24-29. In addition, PM_{10} exposure was significantly associated with pancreatic cancer risk in Latinos only.
Al-Hamdan et al., 2017[96]	USA	Examining the association between exposure to $PM_{2.5}$ and respiratory system cancer incidence in the US population.	Ecological & Spatial Studies		Air Pollution: $PM_{2.5}$, Sociodemographic	Respiratory system cancer	A significant association between $PM_{2.5}$ exposure and Respiratory system cancer incidence was discovered. Furthermore, the risk was significantly associated with the black population.
Bräuner et al., 2015[133]	Denmark	Evaluating the association between long-term exposure to residential radon and the risk for malignant melanoma (MM) and non-	Cohort Study	51,445 (27,050)	Air Pollution: Radon	Skin Cancer (Basal Cell Carcinoma, Squamous Cell Carcinoma, Malignant Melanoma)	Increased radon exposure was significantly associated with increased Basal cell Carcinoma risk, but not for Squamous Cell Carcinoma and Malignant Melanoma.

		melanoma skin cancer.					
Boz et al., 2024[132]	Switzerland	Assessing the relationship between residential radon exposure and melanoma and squamous cell carcinoma incidence.	Cohort Study	1,362,919 (717,761)	Air Pollution: Radon	Skin Cancer (Squamous Cell Carcinoma, Melanoma)	No significant association was discovered for residential exposure and skin cancer. However, a significant association was observed for melanoma among women aged 20-29 years.
Liao Linda et al., 2016[137]	USA	Evaluating the association between occupational lead exposure and cancer incidence at the stomach, lung, kidney, brain, and meninges	Cohort Study	Others:134,829 (73,363); meningioma: 73,363(73,363)	Air Pollution: Lead Exposure	Stomach Cancer, Lung Cancer, Kidney Cancer, Brain Cancer, Meningioma Cancer	Increased exposure to Lead was significantly associated with increased cancer risk for meningioma in women only.
Karzai et al., 2022[93]	USA	Investigating the association of $PM_{2.5}$ exposure and the risk of papillary thyroid cancer.	Case-Control Study	5,970	Air Pollution: $PM_{2.5}$	Papillary Thyroid Cancer	Increased risk of Papillary thyroid cancer was observed to be significantly associated with increased exposure to $PM_{2.5}$.
Crepeau et al., 2023[97]	USA	Investigating the association between $PM_{2.5}$ and Papillary Thyroid Cancer (PTC) and finding out the patient subgroups at the highest risk of PTC diagnosis.	Case-Control Study	8,909	Air Pollution: $PM_{2.5}$, Socioeconomic	Thyroid Cancer	The PTC was significantly higher for older, female, whites, and never smokers. Furthermore, PTC incidence was higher in the high median household income population.
Shala et al., 2023[164]	Netherlands	Evaluating the relationship between benzene and hydrocarbon exposure with bladder cancer risk.	Cohort Study	2,254	Air Pollution: Benzene, Hydrocarbons	Bladder Cancer	Significant associations were discovered for long-term benzene exposure and bladder cancer risk.
Park et al., 2023[112]	South Korea	Investigating the association between PM_{10} exposure and the urologic cancer risk	Cohort Study	231,997 (52,742)	Air Pollution: PM_{10}	Urologic Cancer, Kidney Cancer, Prostate Cancer	Significant association with increased risk of overall urologic cancer, kidney cancer and prostate cancer.
Brown et al., 2024[121]	USA	Evaluating the associations of residential exposure to $PM_{2.5}$ and NO_2 with uterine cancer	Cohort Study	33,417 (33,417)	Air Pollution: $PM_{2.5}$, NO_2	Uterine Cancer	Increased NO_2 was significantly associated with increased uterine cancer risk, especially among participants living in urban areas.
Chen et al.,2025[118]	China	Investigating the association between long-term exposure to multiple air pollutants ($PM_{2.5}$, PM_{10}, NO_2, NO_x, SO_2, Benzene, and O_3) and the risk of	Cohort Study	210,722(0)	Air Pollution: $PM_{2.5}$, PM_{10}, NO_2, NO_x, SO_2, Benzene and O_3	Prostate Cancer	A significant association was discovered for increased prostate cancer risk with increased exposure to $PM_{2.5}$, PM_{10}, NO_2, NO_x, Benzene, and O_3.

		prostate cancer and their joint effect.					
Park et al.,2025[111]	South Korea	Evaluating the association of four pollutants ($PM_{2.5}$, PM_{10}, NO_2, and O_3) and incident lung cancer in the Seoul Metropolitan Area, South Korea.	Cohort Study	2,035,278	Air Pollution: $PM_{2.5}$, PM_{10}, NO_2, and O_3	Lung Cancer	No significant association of increased lung cancer risk with exposure to the pollutants ($PM_{2.5}$, PM_{10}, NO_2, and O_3) was discovered.
Sun et al., 2025[69]	China	Evaluating the association of long-term fine particulate matter ($PM_{2.5}$) exposure with breast cancer incidence in a women cohort from the China Kadoorie Biobank who were initially free of breast cancer.	Cohort Study	281,152 (281,152)	Air Pollution: $PM_{2.5}$	Breast Cancer	Increased significant association of breast cancer incidence was found with increased exposure to $PM_{2.5}$ for female under age of 60 years. Also, the risk was significantly higher in rural population.
Wu et al.,2024[70]	USA	Investigating the association of breast cancer risk with $PM_{2.5}$ exposure, along with examining the impact of race and ethnicity on the cancer risk.	Cohort Study	58,358 (58,358)	Air Pollution: $PM_{2.5}$, Sociodemographic factors: race, and ethnicity	Breast Cancer	Increased significant association of breast cancer incidence was found with increased exposure to $PM_{2.5}$. However, no differences in associations of the risk were discovered with ethnicity and race.
Zhu et al.,2025[50]	China	Evaluating the association between long-term $PM_{2.5}$ exposure and lung cancer incidence in nonsmokers in a Chinese population, and to assess the modifying effect of genetic factors.	Cohort Study	255,829 (232,188)	Air Pollution: $PM_{2.5}$	Lung Cancer	Increased lung cancer risk and mortality were significantly associated with $PM_{2.5}$ exposure in the non-smoking cohort.
Sun et al.,2025[150]	China	Examining the correlation between phthalate esters (PAEs), particulate matter (PM), and Sick Building Syndrome (SBS) in Shanghai, and further assessing the lifetime incremental cancer risk (ILCR) of di(2-ethylhexyl) phthalate (DEHP) for different age groups across various regions in	Risk assessment and modeling study		Air pollution: Phthalates	Specific cancer type not reported	A significant positive correlation was observed between indoor PAEs, PM ($PM_{2.5}$, PM_{10}), and SBS. The ILCR from intake exceeded the U.S. EPA threshold (1×10^{-6}) in most regions ($1.19–1.93\times10^{-6}$), except in North and South China, while ILCR from inhalation ($0.01–0.75\times10^{-6}$) remained below the limit

		China using region-specific exposure parameters.					
Hu et al., 2025[94]	China	Evaluating the association of long-term exposure to multiple pollutants ($PM_{2.5}$, Nitrate, Sulphate, Ammonium, BC, Organic Matter) with oral cancer risk in Fujian, China.	Cohort study		Air Pollution: $PM_{2.5}$, Nitrate, Sulphate, Ammonium, BC, Organic Matter	Oral cancer	Increased oral cancer risks were reported for increased exposure to the considered pollutants.
Ang et al.,2025[139]	China	Investigating the association of long-term air pollution exposures and esophageal cancer, and assessing air pollution exposure impact on different histological subtypes of esophageal cancer.	Cohort Study	444,932 (243,482)	Air Pollution: $PM_{2.5}$, PM_{10}, NO_2, NO_x, SO_2, Benzene	Esophageal Cancer	Significantly increased cancer risk was associated with increased exposure to NO_2, NO_x, SO_2, and benzene.
Ish et al.,2025[136]	USA	Investigating the association between industrial air pollutants and breast cancer incidence in the USA.	Cohort Study	46,150(46,150)	Air Pollution: Industrial Air Pollutants	Breast Cancer	Increased breast cancer risk was observed with increased exposure to nickel compounds and trichloroethylene; however, the overall mixture of the pollutants did not show such associations.
Liu et al., 2025[56]	China	Investigating the long-term trends in lung cancer (LC) and chronic obstructive pulmonary disease (COPD) mortality linked to particulate matter ($PM_{2.5}$) in China and determining the impact of population aging and other contributing factors on changes in mortality rates	Ecological and spatial study		Air Pollution: $PM_{2.5}$	Lung Cancer	Annual 4.11% increment in Lung Cancer mortality and COPD mortality attributable to $PM_{2.5}$ exposure decreased by 1.23% annually.
Xu et al.,2025[101]	China	Evaluating the association of Dietary protein, $PM_{2.5}$, and their interaction with ovarian cancer mortality.	Cohort Study	658(658)	Air Pollution: $PM_{2.5}$	Ovarian cancer	Increased mortality risk was observed in ovarian cancer patients with increased $PM_{2.5}$ exposure.
Zhaio and Zhu, 2025[90]	China	Assessing the increased exposure risk of air pollutants	Cohort Study		Air pollution: $PM_{2.5}$, NO_2, SO_2; Social: Race, Sex	Colorectal cancer	Increased cancer risk with Increased exposure to Pollutants was observed. The risk was higher in urban residents and females.

		with colorectal cancer risk and its variation with sex, race, and pre-existing health condition					
Ahmadi et al.,2025[117]	Canada	Evaluating the association of NO_2 with breast cancer risk	Cohort Study	24,889 (24,889)	Air pollution: NO_2	Breast Cancer	No significant association was observed between NO_2 exposure and Breast Cancer risk
Zehra et al.,2025[74]	India	Assessing the association of particulate matter and cancer risk in women	Risk assessment and modeling study		Air Pollution: PM_{10}, $PM_{2.5}$, UFP	Specific cancer type not reported	The excess lifetime cancer risk associated with $PM_{2.5}$ and UFP exceeded the permissible limit, showing increased cancer risk from the pollutants in Lucknow, India
Xiong et al.,2025[129]	China	Assessing the lung cancer burden associated to residential radon exposure.	Ecological and spatial study		Air Pollution: Radon	Lung Cancer	The global disease burden of radon-associated lung cancer has decreased. However, it was found to be higher in middle and high-latitude nations.
Bellinato et al, 2025[115]	Italy	Evaluating the association between PM_{10} and $PM_{2.5}$ long-term exposure and melanoma risk	Case-control study	2,575 (1,236)	Air Pollution: PM_{10}, $PM_{2.5}$	Melanoma	Significant inverse association of PM_{10} and $PM_{2.5}$ exposure was discovered with Melanoma risks.
Hurbain et al., 2024[182]	USA	Evaluating the association of air toxic cancer risks with race, education, rurality, and income across the United States at the census tract level.	Ecological & Spatial Studies		Air Pollution: air toxic Sociodemographic: Race, Socioeconomic: income, education	Specific cancer type not reported	Census tracts with a higher population of Black and Asian races had higher cancer risks. Higher income and rurality correlated with lower estimated cancer risks. Education showed a weak positive correlation with the cancer risk.
Grineski et al., 2019[183]	USA	Evaluating cancer risks from hazardous air pollution for Arab-American enclaves in the USA.	Ecological & Spatial Studies		Air Pollution hazardous air pollution Socio-demographic: Race	Specific Cancer type not reported	Residents in Arab enclaves had significantly higher cancer risks than those in non-enclaves from the hazardous air pollution.
Ekenga et al., 2019[180]	USA	Evaluating the association of neighborhood isolation and sociodemographic characteristics with exposure to carcinogenic air toxics in St. Louis, USA	Cross-Sectional Study		Air Pollution and Sociodemographic	Specific Cancer type not reported	Non-white population, African Americans, people without a high school education, lower-income households, individuals living in poverty, and the unemployed are all at higher risk of cancer due to exposure to air toxic hotspots in St. Louis, USA.
Jia et al., 2014[181]	USA	Evaluating the association of cancer risks from harmful air toxics and racial composition in Memphis/Shelby County, Tennessee, U.S.A.	Ecological & Spatial Studies		Air Pollution, Sociodemographic: racial composition	Specific Cancer type not reported	The cumulative cancer risks increased with the increase in population density. They were found to be 6 % higher in the African American concentrated census tracts than in the white concentrated tracts, with disparity varying by emission source.

Table S4. Review summary of the publications studying the association of water and land pollution with cancer

Study	Country of origin	Study Objective	Study Design	Total Population (Female Population)	Concerned Contributing Factors	Health outcome	Key Findings
Yadav and Kalkal, 2024[156]	India	Calculating health hazard parameters due to arsenic contamination in groundwater in India	Risk Assessment & Modelling		Water Pollution: Arsenic (As)	Specific Cancer type not reported	Cancer risks from Arsenic Exposure due to groundwater contamination were higher than the suggested USEPA limits of 10^{-4}.
Bekoe et al., 2024[157]	Ghana	Evaluating health risks from exposure to surface waters	Risk Assessment & Modelling		Water Pollution: Lead, Cadmium	Specific Cancer type not reported	Cancer risks from lead were in the acceptable region, whereas those from cadmium were higher than the USEPA acceptable level.
Aendo et al., 2022[158]	Thailand	Evaluating the cancer risks from heavy metals in groundwater due to contamination from municipal solid waste.	Cross-Sectional Study		Water Pollution: Lead (Pb), Cadmium (Cd), Nickel, Chromium	Specific Cancer type not reported	The cancer risks from lead, cadmium, nickel, and chromium were higher than the USEPA carcinogenic risk limit.
Ahmed et al., 2021[159]	Malaysia	Evaluating the carcinogenic risk of arsenic contamination in the drinking water supply chain of the Langat River basin.	Risk Assessment & Modelling		Water Pollution: Arsenic (As)	Specific Cancer type not reported	The arsenic contamination in the assessed region showed no significant cancer risk.
Nyambura et al., 2020[160]	Kenya	Evaluating the Carcinogenic risks for Cadmium, Nickel, and lead from water consumption in the Kilimambogo region of Kenya.	Ecological & Spatial Studies		Water Pollution: Cadmium, Nickel, Lead	Specific Cancer type not reported	The cancer risks from lead, cadmium, and nickel were higher than the USEPA carcinogenic risk limit in the Kilimambogo region.
Tsai et al., 2021[161]	Taiwan	Investigating the relationship between arsenic exposure and the	Case-Control Study	788 (386)	Water Pollution: Arsenic	Bladder cancer, Kidney cancer	High arsenic exposure significantly increased the risk of bladder cancer, but that for

		incidence of kidney and bladder cancers.					kidney cancer was not statistically significant.
Hasan et al., 2024[162]	USA	Evaluating the association of arsenic in drinking water and kidney cancer in Texas counties.	Ecological & Spatial Studies	28,896	Water Pollution: Arsenic	Kidney cancer	A significant relation was discovered for medium (1-5ppb) and high (>5ppb) arsenic exposure and kidney cancer.
Li et al., 2022[163]	Sweden	Evaluating the cancer risk associated with polyfluoroalkyl substances (PFAs) in drinking water	Cohort Study	60,507 (27,569)	Water Pollution: PFAs	Overall cancer, Stomach cancer, Colon cancer, Rectum cancer, Gall bladder cancer, bile duct cancer, Pancreas cancer, Trachea cancer, lung cancer, Breast cancer, Cervix cancer, Uterus cancer, Ovarian cancer, Prostate cancer, Testicle cancer, Kidney cancer, Bladder cancer, Skin melanoma cancer, Skin non-melanoma cancer, Brain cancer, Thyroid cancer, Bone cancer, cartilage cancer, non-Hodgkin lymphoma, Myeloma, Chronic lymphocytic leukemia	No significant associations were discovered in the study.
Cui et al., 2017[164]	China	Assessing the relation between cancer risks and PCP exposure in a community population along the Yangtze River in China	Ecological & Spatial Studies	15,962 (5,967)	Water Pollution: Pentachlorophenol (PCP)	Esophagus Cancer, Stomach Cancer, Colon Cancer, Rectum Cancer, Anus Cancer, Liver Cancer, Gallbladder Cancer, Pancreas Cancer, Tracheal Cancer, Bronchus Cancer, Lung Cancer, Breast Cancer, Uterus Cancer, Ovary Cancer, Prostate Cancer, Cerebrum Central Nervous Cancer, Thyroid Cancer, Lymph Cancer, and Leukemia	Significant positive associations were discovered with multiple cancers (lymph, Blood, nasopharynx, gall bladder, pancreas, liver, and respiratory system) for PCP exposure.
Roh et al., 2017[165]	USA	Estimating the risk associated with prostate cancer from arsenic exposure from drinking water.	Ecological & Spatial Studies		Water Pollution: Arsenic	Prostate Cancer	A significant risk ratio of prostate cancer was observed in counties with medium-level and high arsenic levels in drinking water.

Nde et al., 2021[166]	South Africa	Evaluating the carcinogenic risks from the consumption of crops grown in contaminated land	Cross-Sectional Study		Land Pollution: Nickel (Ni), Chromium (Cr)	Specific Cancer type not reported	The consumption of crops grown on contaminated land increased cancer risks.
Tse et al., 2018[167]	Hong Kong	Assessing the association of Bisphenol A (BPA) exposure and prostate cancer risk	Case-Control Study	833	Other Pollution: BPA	Prostate Cancer	BPA exposure was significantly associated with prostate cancer risk, especially in men under the age of 70.
Akinipe et al.,2025[168]	Nigeria	Investigating the radiological health hazard (the annual effective dose (AED) and the excess lifetime cancer risk (ELCR)) associated to radon in various water sources at Redeemer's University campus, Ede, and its environment.	Risk assessment and modelling study		Water Pollution: Radon	Specific cancer type not reported	The annual effective dose (AED) and the excess lifetime cancer risk (ELCR) from radon were below the desired limit levels.

Table S5: Data used for meta-analyses

Study ID	Cancer	Exposure variable	Effect Type	Effect size	LCL	UCL	SE
[36]	Bladder Cancer	$PM_{2.5}$	HR	1.61	1.5	1.72	0.0349
[174]	Bladder Cancer	$PM_{2.5}$	HR	1.09	0.93	1.27	0.0795
[175]	Bladder Cancer	$PM_{2.5}$	HR	0.96	0.71	1.31	0.1563
[49]	Bladder Cancer	$PM_{2.5}$	HR	1.05	0.62	1.79	0.2705
[34]	Blood Cancer (Leukemia)	$PM_{2.5}$	HR	1.06	0.88	1.27	0.0936
[72]	Blood Cancer (Leukemia)	$PM_{2.5}$	HR	1.58	0.87	2.88	0.3054
[80]	Blood Cancer (Leukemia)	$PM_{2.5}$	HR	0.91	0.79	1.05	0.0726
[120]	Blood Cancer (Leukemia)	$PM_{2.5}$	HR	1	0.84	1.18	0.0867
[75]	Malignant Brain Tumors	$PM_{2.5}$	HR	1.049	0.747	1.476	0.1737
[77]	Malignant Brain Tumors	$PM_{2.5}$	HR	1.27	0.49	3.3	0.4865
[79]	Malignant Brain Tumors	$PM_{2.5}$	HR	1.04	0.66	1.63	0.2306
[5]	Malignant Brain Tumors	$PM_{2.5}$	HR	0.95	0.22	4.2	0.7524
[58]	Breast Cancer	NO_2	HR	1	0.97	1.03	0.0153
[59]	Breast Cancer	NO_2	HR	1.81	1.51	2.17	0.0925
[60]	Breast Cancer	NO_2	HR	0.99	0.92	1.05	0.0337

[61]	Breast Cancer	NO_2	HR	1.09	0.91	1.31	0.0929
[62]	Breast Cancer	NO_2	HR	1.02	0.98	1.06	0.0200
[63]	Breast Cancer	NO_2	HR	1.02	0.97	1.07	0.0250
[32]	Breast Cancer	NO_2	HR	1.08	1.03	1.13	0.0236
[113]	Breast Cancer	NO_2	HR	1.03	0.97	1.1	0.0321
[67]	Breast Cancer	NO_2	HR	0.99	0.97	1.01	0.0103
[58]	Breast Cancer	PM_{10}	HR	1.99	1.75	2.27	0.0664
[61]	Breast Cancer	PM_{10}	HR	1.05	0.95	1.16	0.0509
[62]	Breast Cancer	PM_{10}	HR	1.08	0.9	1.28	0.0899
[63]	Breast Cancer	PM_{10}	HR	0.99	0.98	1	0.0052
[64]	Breast Cancer	PM_{10}	HR	1.01	0.97	1.05	0.0202
[113]	Breast Cancer	PM_{10}	HR	1.04	0.95	1.14	0.0465
[65]	Breast Cancer	PM_{10}	HR	0.98	0.92	1.04	0.0313
[36]	Breast Cancer	$PM_{2.5}$	HR	1.54	1.49	1.61	0.0198
[58]	Breast Cancer	$PM_{2.5}$	HR	0.86	0.66	1.12	0.1349
[59]	Breast Cancer	$PM_{2.5}$	HR	1.14	0.95	1.38	0.0952
[60]	Breast Cancer	$PM_{2.5}$	HR	0.97	0.92	1.02	0.0263
[61]	Breast Cancer	$PM_{2.5}$	HR	1.1	0.85	1.42	0.1309
[62]	Breast Cancer	$PM_{2.5}$	HR	0.99	0.73	1.33	0.1530
[63]	Breast Cancer	$PM_{2.5}$	HR	1.03	0.96	1.11	0.0370
[32]	Breast Cancer	$PM_{2.5}$	HR	1.05	1	1.11	0.0266
[64]	Breast Cancer	$PM_{2.5}$	HR	1.03	0.94	1.13	0.0470
[65]	Breast Cancer	$PM_{2.5}$	HR	0.89	0.78	1.01	0.0659
[33]	Breast Cancer	$PM_{2.5}$	HR	1.02	1.01	1.03	0.0050
[66]	Breast Cancer	$PM_{2.5}$	HR	1.07	1.01	1.14	0.0309
[67]	Breast Cancer	$PM_{2.5}$	HR	1	0.99	1.02	0.0076
[71]	Lung Cancer	Black Carbon	HR	1.19	0.87	1.63	0.1602
[39]	Lung Cancer	Black Carbon	HR	1.1	1.05	1.16	0.0254
[44]	Lung Cancer	Black Carbon	HR	1.08	1.02	1.13	0.0261
[49]	Lung Cancer	Black Carbon	HR	1.47	1.08	2.02	0.1597
[71]	Lung Cancer	NO_2	HR	1.06	0.88	1.27	0.0936
[67]	Lung Cancer	NO_2	HR	0.99	0.97	1	0.0078
[37]	Lung Cancer	NO_2	HR	1.22	1.14	1.3	0.0335
[106]	Lung Cancer	NO_2	HR	0.96	0.96	1.09	0.0324

[39]	Lung Cancer	NO_2	HR	1.1	1.05	1.15	0.0232
[42]	Lung Cancer	NO_2	HR	1.24	1.05	1.47	0.0858
[57]	Lung Cancer	NO_2	HR	0.92	0.74	1.15	0.1125
[44]	Lung Cancer	NO_2	HR	1.09	1.04	1.16	0.0279
[46]	Lung Cancer	NO_2	HR	1.013	0.012	1.013	1.1316
[49]	Lung Cancer	NO_2	HR	1.44	1.02	2.05	0.1781
[67]	Lung Cancer	O_3	HR	0.96	0.95	0.97	0.0053
[39]	Lung Cancer	O_3	HR	0.82	0.77	0.88	0.0341
[44]	Lung Cancer	O_3	HR	0.91	0.86	0.96	0.0281
[46]	Lung Cancer	O_3	HR	0.991	0.99	0.992	0.0005
[36]	Lung Cancer	$PM_{2.5}$	HR	1.65	1.56	1.75	0.0293
[71]	Lung Cancer	$PM_{2.5}$	HR	1.01	0.68	1.51	0.2035
[67]	Lung Cancer	$PM_{2.5}$	HR	0.99	0.98	1	0.0052
[37]	Lung Cancer	$PM_{2.5}$	HR	1.28	1.21	1.36	0.0298
[106]	Lung Cancer	$PM_{2.5}$	HR	0.98	0.53	1.81	0.3133
[39]	Lung Cancer	$PM_{2.5}$	HR	1.21	1.11	1.31	0.0423
[40]	Lung Cancer	$PM_{2.5}$	HR	1.43	1.03	2	0.1693
[41]	Lung Cancer	$PM_{2.5}$	HR	1.47	1.22	1.77	0.0949
[107]	Lung Cancer	$PM_{2.5}$	HR	1.2	0.97	1.49	0.1095
[42]	Lung Cancer	$PM_{2.5}$	HR	1.12	0.89	1.4	0.1156
[108]	Lung Cancer	$PM_{2.5}$	HR	1.05	0.9	1.23	0.0797
[57]	Lung Cancer	$PM_{2.5}$	HR	0.85	0.53	1.36	0.2404
[48]	Lung Cancer	$PM_{2.5}$	HR	2.58	1.84	3.63	0.1733
[49]	Lung Cancer	$PM_{2.5}$	HR	1.95	1.42	2.68	0.1620
[37]	Lung Cancer	PM_{10}	HR	1.07	1.02	1.13	0.0261
[106]	Lung Cancer	PM_{10}	HR	1.16	0.89	1.51	0.1349
[107]	Lung Cancer	PM_{10}	HR	1.23	1.04	1.46	0.0865
[108]	Lung Cancer	PM_{10}	HR	1.06	0.98	1.16	0.0430
